# Genome-Wide Analysis of PAPS1-Dependent Polyadenylation Identifies Novel Roles for Functionally Specialized Poly(A) Polymerases in *Arabidopsis thaliana*

**DOI:** 10.1371/journal.pgen.1005474

**Published:** 2015-08-25

**Authors:** Christian Kappel, Gerda Trost, Hjördis Czesnick, Anna Ramming, Benjamin Kolbe, Son Lang Vi, Cláudia Bispo, Jörg D. Becker, Cornelia de Moor, Michael Lenhard

**Affiliations:** 1 Universität Potsdam, Institut für Biochemie und Biologie, Potsdam-Golm, Germany; 2 Instituto Gulbenkian de Ciência, Oeiras, Portugal; 3 University of Nottingham, School of Pharmacy, Centre for Biomolecular Sciences, University Park, Nottingham United Kingdom; The University of North Carolina at Chapel Hill, UNITED STATES

## Abstract

The poly(A) tail at 3’ ends of eukaryotic mRNAs promotes their nuclear export, stability and translational efficiency, and changes in its length can strongly impact gene expression. The *Arabidopsis thaliana* genome encodes three canonical nuclear poly(A) polymerases, PAPS1, PAPS2 and PAPS4. As shown by their different mutant phenotypes, these three isoforms are functionally specialized, with PAPS1 modifying organ growth and suppressing a constitutive immune response. However, the molecular basis of this specialization is largely unknown. Here, we have estimated poly(A)-tail lengths on a transcriptome-wide scale in wild-type and *paps1* mutants. This identified categories of genes as particularly strongly affected in *paps1* mutants, including genes encoding ribosomal proteins, cell-division factors and major carbohydrate-metabolic proteins. We experimentally verified two novel functions of *PAPS1* in ribosome biogenesis and redox homoeostasis that were predicted based on the analysis of poly(A)-tail length changes in *paps1* mutants. When overlaying the PAPS1-dependent effects observed here with coexpression analysis based on independent microarray data, the two clusters of transcripts that are most closely coexpressed with *PAPS1* show the strongest change in poly(A)-tail length and transcript abundance in *paps1* mutants in our analysis. This suggests that their coexpression reflects at least partly the preferential polyadenylation of these transcripts by PAPS1 versus the other two poly(A)-polymerase isoforms. Thus, transcriptome-wide analysis of poly(A)-tail lengths identifies novel biological functions and likely target transcripts for polyadenylation by PAPS1. Data integration with large-scale co-expression data suggests that changes in the relative activities of the isoforms are used as an endogenous mechanism to co-ordinately modulate plant gene expression.

## Author Summary

The poly(A) tail of eukaryotic mRNAs promotes export from the nucleus, translation in the cytoplasm and stability of the mRNA, and changes in poly(A)-tail length can strongly impact on gene expression. The *Arabidopsis thaliana* genome encodes three nuclear canonical poly(A) polymerases (PAPS1, PAPS2, PAPS4) that fulfill different functions, presumably by preferentially polyadenylating certain subpopulations of pre-mRNAs. Here, we use a fractionation-based technique to assess the transcriptome-wide impact of reduced PAPS1 activity and identify functional classes of transcripts that are particularly sensitive to reduced PAPS1 activity. Analysis of these transcripts identifies two novel biological functions for *PAPS1* in ribosome biogenesis and in redox homeostasis that we confirm experimentally. By overlaying our results with information about genome-wide co-expression, we demonstrate that genes co-expressed with *PAPS1* are the most strongly affected in terms of poly(A)-tail length and total-abundance changes in the *paps1* mutants. This provides strong evidence that the co-expression of these genes with *PAPS1* that is seen across thousands of microarrays is at least partly caused by altered activity of the PAPS1 isoform, suggesting that the plant indeed uses modulation of the balance of isoform activities to coordinately regulate the expression of groups of genes.

## Introduction

The poly(A) tail is an essential modification found at the 3’ ends of virtually all eukaryotic mRNAs [1,2,3]. After transcription of the pre-mRNA, sequences in the 3’ UTR recruit two protein complexes, Cleavage and Polyadenylation Specificity Factor (CPSF) and Cleavage Stimulation Factor (CStF) that effect endonucleolytic cleavage of the pre-mRNA, exposing a 3’-OH end, and recruit poly(A) polymerase to synthesize the poly(A) tail [1,2,3]. The poly(A) tail serves three major functions: promoting nuclear export of the mRNA, stimulating efficient translation, and stabilizing the mRNA in the cytosol. The poly(A) tail has been reported to channel transcripts into the dedicated mRNA export pathway [4,5], and in yeast this effect appears to be mediated by the poly(A)-binding protein Pab1 [6]. The poly(A) tail stimulates translation of the mRNA by promoting a close contact between the 3’ and 5’ ends of the mRNA and thus promotes efficient translational initiation [7,8]. This is mediated by interactions between the cytoplasmic poly(A)-binding protein PABPC bound to the poly(A) tail and translation initiation factors, in particular eIF4G, bound to the 5’ cap. A correlation has been observed between poly(A)-tail length and translational stimulation, i.e. long poly(A) tails promote translation more strongly than shorter ones, suggesting that modulation of poly(A)-tail length could be used to regulate the efficiency of translation [9,10]. The third major function of the poly(A) tail relates to mRNA stability [1]. For the bulk of cellular mRNAs, deadenylation, i.e. shortening of the poly(A) tail from the 3’ end, down to an oligo(A) tail is a pre-requisite for degradation, triggering decapping at the 5’ end and subsequent 5’-to-3’ exonucleolytic degradation or, less commonly, 3′-to-5′ degradation by the exosome [11,12,13,14]. Most mRNAs in yeast and mammals are believed to start out with a rather uniform length of the poly(A) tail of around 70–80 and 250 As, respectively, and deadenylation occurs with transcript-specific rates [12,15]. This rate of deadenylation is therefore a major determinant of transcript half-life. An additional factor is the stability of the oligoadenylated form of the transcript before decapping and degradation, which appears to differ between mRNAs [12]. The combination of the kinetics of deadenylation and the stability of the oligoadenylated form determines not only the stability of the transcript, but also the average poly(A) tail length of an mRNA in steady-state conditions. Thus, large-scale studies of poly(A)-tail length in mammalian cells have identified a number of very stable transcripts with only very short poly(A) tails [16,17], likely reflecting a high stability of the oligoadenylated state.

Control of gene expression via modulation of poly(A)-tail length plays a prominent role during germ-cell and early embryo development in *Drosophila melanogaster*, *Caenorhabditis elegans* and vertebrates [8,18,19,20,21,22,23,24,25]. In vertebrates, maternally deposited mRNAs in the egg cytoplasm are only translated after fertilization when their poly(A) tails are extended by the action of cytoplasmic poly(A) polymerase [8]. The importance of translational regulation by modulating poly(A)-tail length appears to change during animal embryonic development. In particular, a positive correlation between poly(A)-tail lengths and translational efficiency is seen in early embryos, but not in later stages of development [17]. In contrast to cytoplasmic modulation of poly(A)-tail length, initial polyadenylation of pre-mRNAs in the nucleus is thought to not be used for regulating gene expression on a larger scale [1]. Although a non-canonical poly(A) polymerase in mammals, Star-PAP, is required specifically for polyadenylation of certain pre-mRNAs encoding proteins required for the response to oxidative stress [26], the bulk of pre-mRNAs appears to be appended with a standard length of poly(A) tail by canonical nuclear poly(A) polymerases [1]. In mammals, where this initial length control is particularly well understood, the length of 250 As results from an interaction between CPSF and the nuclear poly(A) binding protein PAPBN tethering poly(A) polymerase to the 3’ end, which can only be maintained for poly(A) tails of less than about 250 As; a longer tail cannot be accommodated, causing poly(A) polymerase to revert to its distributive mode of action and to ultimately dissociate from the 3’ end of the transcript [1,27,28].

The *Arabidopsis thaliana* genome encodes four isoforms of canonical poly(A) polymerase, of which PAPS1, PAPS2 and PAPS4 are located in the nucleus and are expressed widely in the plant in a largely overlapping pattern, while PAPS3 is a cytoplasmic protein expressed mainly in pollen [29,30]. Recent mutant analysis of the nuclear isoforms has uncovered functional specificity amongst them. PAPS1 activity is essential for male gametophyte function, and reduced activity in the sporophyte causes impaired leaf growth, a constitutive immune response, and overgrowth of floral organs; by contrast, even combined loss of PAPS2 and PAPS4 function does not interfere with gametophyte or sporophyte viability, and double mutants do not show any obvious morphological defects [31,32]. Notably, this is not due to PAPS1 simply being responsible for the bulk of pre-mRNA polyadenylation, as even a severe reduction in PAPS1 activity has virtually no effect on bulk poly(A)-tail lengths [32]. Rather, it suggests that there are some pre-mRNAs that are preferentially polyadenylated by PAPS1 and whose misexpression in *paps1* mutants causes their specific mutant phenotypes. These specific PAPS1 targets include mRNAs of the *SMALL AUXIN UP RNA* (*SAUR*) family, whose poly(A) tail is strongly shortened in *paps1* mutants and whose reduced activity appears to contribute to the growth defect of the mutant leaves.

To obtain a transcriptome-wide view of the changes in poly(A)-tail length that result from altered PAPS1 activity, we employed an mRNA fractionation method followed by RNA-seq [16]. Transcripts affected in poly(A)-tail length in *paps1* mutants are enriched for ribosomal protein-encoding mRNAs and ones linked to plastid redox status, and *paps1* mutants indeed show defective ribosome content, altered plastid redox status and a higher resistance to reactive-oxygen species, which is not seen in *paps2 paps4* mutants. Molecular and double-mutant analysis indicates that the activities of PAPS1 and the CPSF subunit OXT6 interact in the oxidative-stress response. Overlaying the changes in poly(A)-tail lengths with information on coexpression of genes provides evidence that changes in the activity level of PAPS1 contribute to altered gene expression in response to environmental or internal cues.

## Results

### Fractionation of mRNA based on poly(A)-tail length

**Fig 1:**
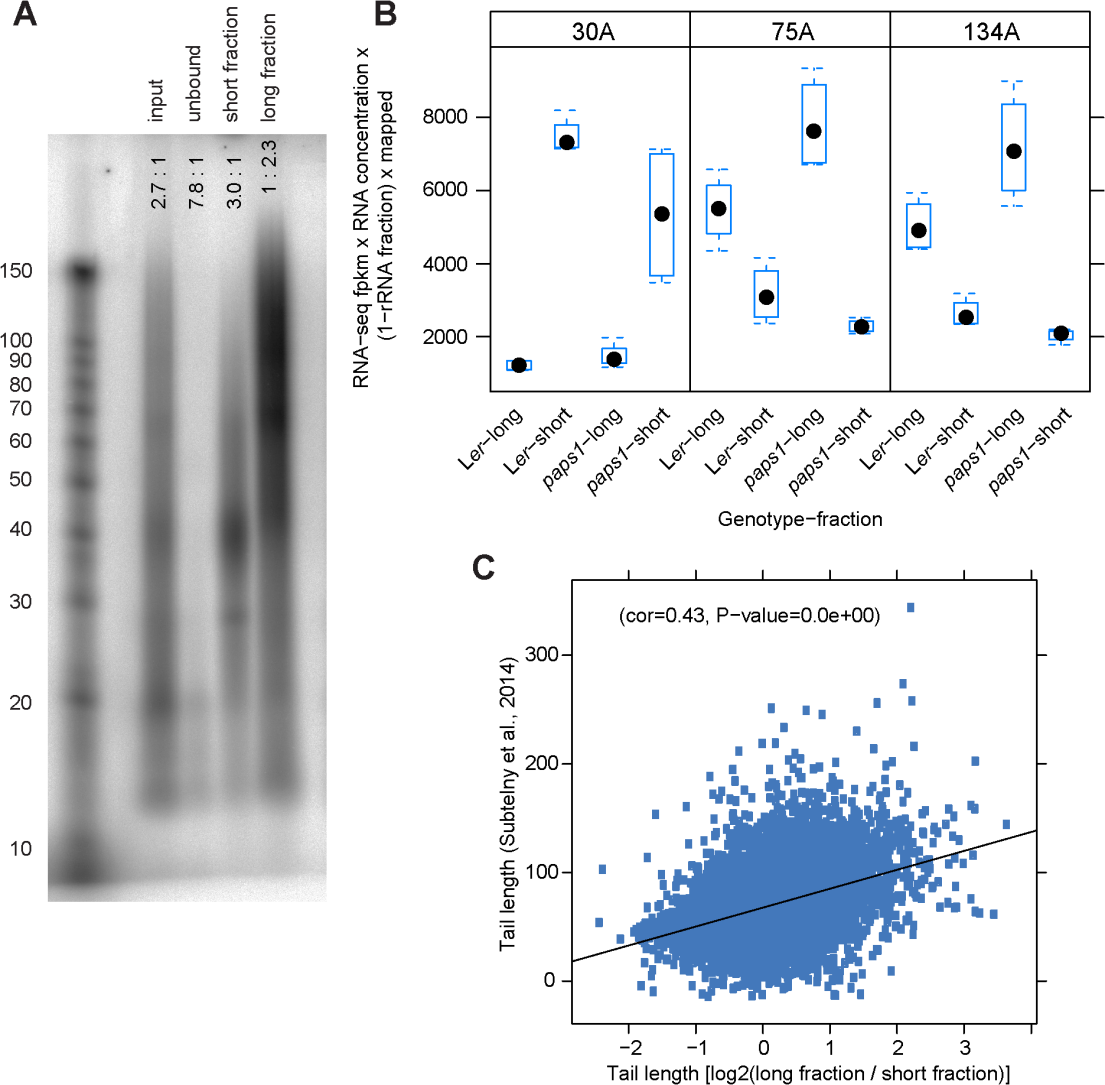
A fractionation-based approach to determine transcriptome-wide poly(A)-tail lengths. (A) Bulk poly(A)-tail analysis of the input and the unbound, short-tail and long-tail fractions from wild-type seedlings. RNA was 3’ labeled with ^32^P-cordycepin and yeast poly(A) polymerase, before digesting all non-poly(A) RNA using RNAse A and RNAse T1, which specifically hydrolyze RNA at C and U residues (RNAse A) and at G residues (RNAse T1). The remaining ^32^P-labeled poly(A) tails were separated by urea-PAGE before Phosphorimager analysis. Fragment sizes in nucleotides are indicated on the left based on a labeled RNA-size marker. Values indicated on top of the lanes are ratios of radioactive signal above 50 nucleotides to radioactive signal below 50 nucleotides in the respective lane. A replicate experiment is shown in S1A Fig. (B) Abundance estimates of the three spike-in control RNAs from RNA-seq of the short- and long-tail fractions in L*er* wild-type and *paps1-1* mutants. Box-plots show the distribution of values from four biological replicates each. Filled black dots indicate the median values. (C) Correlation of poly(A)-tail length estimates of wild-type plants obtained here with those from a previous study based on PAL-seq [17].

To obtain a genome-wide view of the transcripts whose polyadenylation depends on PAPS1 activity, we estimated poly(A)-tail lengths by using an established fractionation method [16] combined with RNA-seq. Total RNA is hybridized to an excess of biotinylated (dT)_17_ oligonucleotide and captured on streptavidin beads. After washing, a low-stringency elution recovers those transcripts with only a short poly(A) tail (in our case ones with less than approximately 50 As), before a second elution step recovers the remaining transcripts with longer poly(A) tails (Figs 1A and S1A). The paired fractions obtained from the starting samples of total RNA were then subjected to RNA-seq to quantify transcript abundances, using 50-bp single-end reads (S1 Table). To abolish PAPS1 activity as far as possible, while still allowing for the generation of sufficient material for analysis, we used the *paps1-1* mutant that encodes a PAPS1 protein that is inactivated at high temperatures [32]. After growth at 21°C, L*er* wild-type and *paps1-1* mutant seedlings were shifted to 28°C for two hours before harvesting to abolish the activity of the mutant protein. In the absence of information about the kinetics of deadenylation of PAPS1-target transcripts, and given a maximum poly(A)-tail length of around 150–200 As in *A*. *thaliana* (Fig 1A), we chose a cut-off of around 50 As for the fractionation rather than a lower value, in order to increase the chances of detecting tail-length changes between wild-type and mutant also for more slowly deadenylated PAPS1-target transcripts. Four biological replicates per genotype were used. As internal controls for the fractionation, we generated three synthetic RNAs with defined poly(A)-tail lengths of 30 A, 75 A and 134 A by *in vitro* transcription and added equal amounts of these to each of the eight starting total-RNA samples. After aligning the sequence reads to the *Arabidopsis* reference genome, fpkm (fragments per kilobase of exon per million fragments mapped) values were calculated for each transcript as a measure of its relative abundance in the fraction. The ratio of transcript abundance in the fraction with short poly(A) tails and that with long poly(A) tails (termed the short and long fractions from here on) was then determined as a proxy for the distribution of poly(A)-tail lengths on each transcript using edgeR (see Methods). The relatively high percentage of reads corresponding to rRNA (S1 Table) likely reflects the presence of oligoadenylated pre-rRNA and rRNA molecules as intermediates in pre-rRNA surveillance and rRNA degradation [33,34]; contamination with these reads results from the fractionation protocol requiring only relatively mild washes so as not to lose a large fraction of the mRNAs with short poly(A) tails. However, as the proportion of these reads is comparable across the different ‘long’ and ‘short’ fractions, it is unlikely to distort the analysis of the mRNA reads.

We technically validated the fractionation and the RNA-seq based estimates of transcript abundances in five ways. First, we performed qRT-PCR against the three spike-in control RNAs on identical-volume aliquots of the 16 fractions (S1B and S1C Fig). We used individual PCR efficiency to the power of-c_t_ (PCReff^-ct^) as an estimate of abundance in the absence of any reference. As expected, the 30A control was about 2.4 (wild type) and 1.3 (*paps1*) times more abundant in the short than in the long fraction. By contrast, the 75A and 134A fractions were about 4.9 (wild type), 6.2 (*paps1*) and 4.4 (wild type), 7.9 (*paps1*) times more abundant in the long fractions, respectively (S1C Fig).

Second, an analogous estimate was obtained for the three spike-in controls from the RNA-seq data. Relative transcript abundances of the three RNAs as expressed by fpkm values were multiplied with the overall RNA concentrations in the sixteen samples and corrected for the proportion of non-rRNA reads that could be mapped to obtain a proxy for their absolute abundance in each of the sixteen fractions. This estimate should then be comparable to the qRT-PCR measurements above. The 30A control RNA was about 6.1 (wild type) and 3.1 (*paps1*) times more abundant in the short fraction, while the 75A and 134A controls about 1.7 (wild type), 3.4 (*paps1*) and 1.9 (wild type), 3.5 (*paps1*) times more abundant in the long fractions (Fig 1B). We note that both estimates for the spike-in controls indicated that for the *paps1-1* samples relatively more of the 30A control RNA was seen in the long fraction, while relatively less of the 75A and 134A controls was found in the short fractions compared to wild-type. This suggested that despite processing wild-type and mutant samples in parallel, there was variation in the fractionation for unknown reasons; as this variation globally affects the absolute values of the long/short ratios for all transcripts, our analysis of *PAPS1*-specific effects (see below) accounts for this variation by considering the behaviour of groups of genes relative to the respective transcriptome-wide background (i.e. all other transcripts not belonging to the focal group). This approach is thus independent of the absolute values of the long/short-ratios. In addition this group-wise analysis mitigates the effect of technical variation in individual transcript measurements.

Third, we determined the abundances of 15 endogenous transcripts chosen based on preliminary analyses in the 16 fractions by qRT-PCR (as PCReff^-ct^ on identical-volume aliquots) and correlated these with their fpkm values from RNA-seq (S2A Fig). For 13 out of the 16 fractions these values were significantly correlated with Pearson correlation coefficients between 0.61 and 0.85; in the remaining three fractions, there was one strong outlier each from the genes with very low RNA-seq expression estimates. Fourth, a PCR-based assay, the so-called ePAT test [35], was used to independently determine changes in poly(A)-tail length between mutant and wild-type samples (S3 Fig). Out of 40 transcripts with a predicted change in poly(A)-tail length that were selected for validation, we were able to design primers allowing robust and specific amplification for 15 transcripts. Of these, 11 showed evidence for a shorter poly(A)-tail in *paps1* mutants, in accordance with the prediction based on RNA-seq, while for three transcripts there was no change and one transcript appeared to have a longer poly(A) tail in the mutant (S3 Fig); the latter four transcripts tended to have lower predicted fold-changes of poly(A)-tail length than the 11 transcripts with successful validation. Fifth, we compared our estimates of poly(A)-tail length with those from a recent transcriptome-wide study that used the novel PAL-seq method on mature *A*. *thaliana* leaves [17]. Our estimates are in good agreement with the published values (Fig 1C; Pearson correlation coefficient 0.43).

Thus, we conclude that our fractionation successfully resolved different populations of transcripts based on their poly(A)-tail lengths; that the fpkm values determined by RNA-seq can be used as estimates for the relative abundances of the transcripts in the two fractions; and that the edgeR ratios can be used as proxies for the poly(A)-tail length distributions of the transcripts. We excluded transcripts with low abundance (five or fewer reads in one or more samples) from our analyses, as the samples did not cluster by genotype when including all transcripts. When applying the filter, the samples clustered as expected based on their long/short-ratios, i.e. their estimated poly(A)-tail lengths (S1D Fig).

### Correlations between poly(A)-tail length and other transcript features in wild type

We next asked whether correlations between poly(A)-tail length and other transcript features observed in other systems are also seen in *Arabidopsis*. To this end, we related our estimates of poly(A)-tail length in wild type to total transcript abundance, features of the 3’ UTR and transcript stability. Total transcript abundance in seedlings was estimated from independent publicly available transcriptomic experiments using Illumina sequencing (see Methods). The length of the annotated 3’ UTR and the number of annotated 3’ UTRs, i.e. the number of different 3’ cleavage sites detected for a given transcript were based on combined information from TAIR10 annotation (www.arabidopsis.org) and recent large-scale sequencing efforts of RNA 3’ ends [36,37]. Transcript stability has been determined on a genome-wide basis in *Arabidopsis thaliana* protoplasts [38].

**Fig 2:**
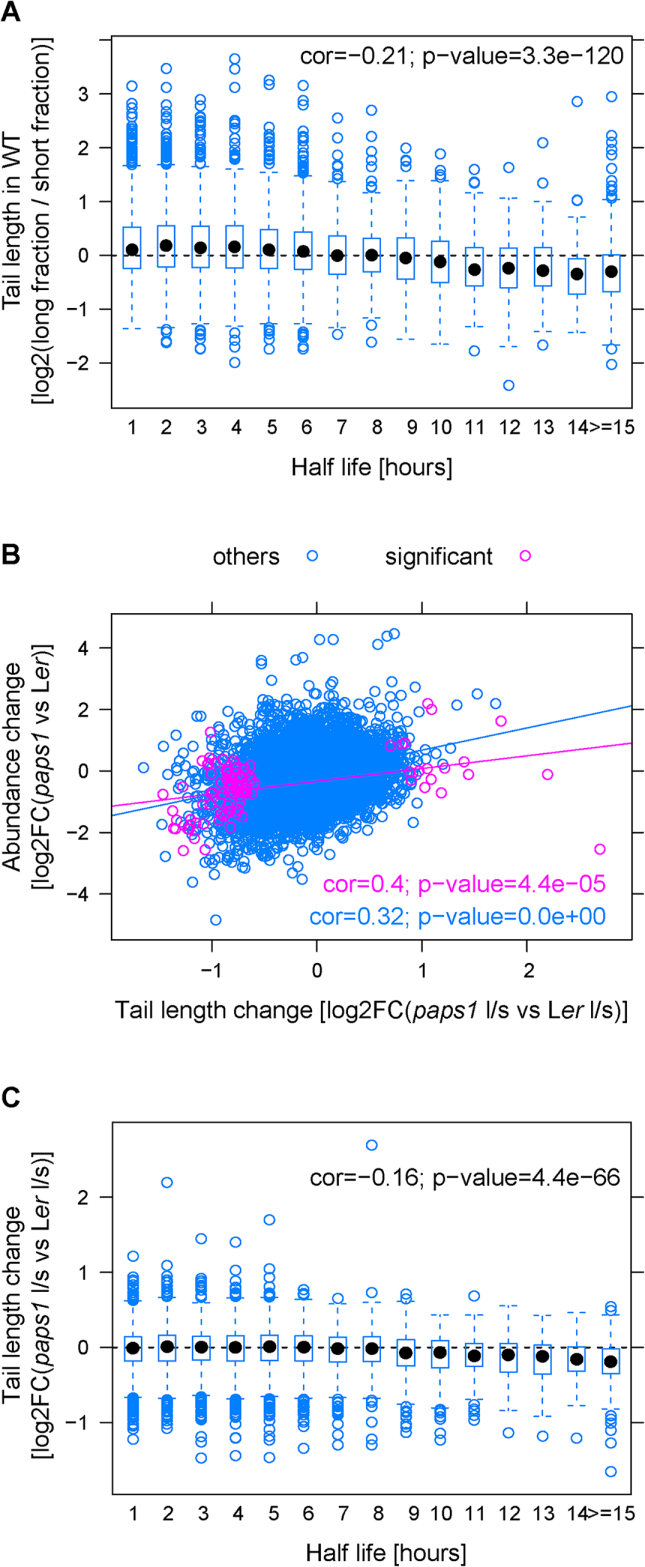
Correlations of mRNA features with poly(A)-tail length in wild type and with the extend of poly(A)-tail length change in *paps1* mutants versus wild type. (A) Correlation of estimates of poly(A)-tail length in wild-type seedlings with the mRNA half-life. Pearson correlation coefficient and p-value is given. (B,C) Correlations of estimates of the change of poly(A)-tail length in *paps1-1* mutants versus wild type with the estimated change in total transcript abundance between the two genotypes (B), and the mRNA half-life (C). Purple symbols in (B) represent the transcripts with a significant tail-length change after multiple-testing correction; purple font in (B) shows the correlation between tail-length and abundance change for the 97 significant transcripts, blue font for all transcripts.

All four correlations of the estimated poly(A)-tail length with transcript abundance, length of the 3’ UTR (expressed as maximum distance between stop-codon and cleavage site, including potential introns), number of annotated or detected 3’ UTRs, and transcript stability were statistically significant (Figs 2A, S4A, S4B and S4C). However, the strength of the correlations, as determined by Pearson correlation coefficient, was very weak for transcript abundance and length and number of 3’ UTRs (S4A, S4B and S4C Fig), questioning their biological significance. By contrast, the measured half-life values showed a moderate negative correlation with our estimates of poly(A)-tail length (Pearson correlation coefficient -0.21; Fig 2A), i.e. mRNAs with a long half-life tended to have a shorter poly(A) tail.

### Functional-category analysis of wild-type poly(A)-tail lengths

We next asked whether gene families or functional categories (as implemented in MapMan) differ in the distribution of their poly(A)-tail lengths in wild type. To this end, we compared the poly(A)-tail length distributions of genes associated with a given functional-category term with the transcriptome-wide background using Wilcoxon rank-sum tests. This identified ribosomal-protein genes, cell-wall proteins and seed storage/lipid-transfer proteins as the functional-category terms with the most significant enrichment for short-tail transcripts and protein post-translational modification (in particular kinases), regulation of transcription, and DNA repair as those associated with the most significant enrichment for long-tail transcripts (S2 Table). We note that transcripts in the ‘short-tail’ class as defined here are presumably still translated efficiently, given that our cut-off for the fractionation of around 50 As is still in the range of poly(A)-tail lengths that support efficient translational initiation via formation of a closed-loop structure [39].

### Reduction of PAPS1 function changes poly(A)-tail lengths of selected gene classes

In order to identify changes in poly(A)-tail length resulting from reduced PAPS1 function, the long/short-ratios for individual genes were compared between the four wild-type and the four mutant samples using R/Bioconductor package edgeR. After correcting for multiple testing, only 97 transcripts showed a significant change in their long/short ratios between the two genotypes (S3 Table and Fig 2B). This suggests that the technical variation in the measurements (see above) renders the analysis of individual transcripts less meaningful. To circumvent this issue, we focussed on gene families and functional MapMan categories and compared the long/short-ratios of members of the group with the transcriptome-wide background using Wilcoxon rank-sum tests to detect significant differences at the family or category levels (S4 Table). Validating our approach, the family of *SAUR* transcripts showed a significant shortening of poly(A) tails in *paps1-1* mutants compared to the transcriptome-wide background (p<0.001), as was predicted from our previous analysis of individual *SAUR* family members by a PCR-based poly(A)-tail length assay [32]. By far the most significantly changed category with shorter tails in the mutants were genes encoding ribosomal proteins (p = 5.1E-43), followed by genes encoding histones (p = 1.8E-05) and pectin-methylesterase inhibitors (9.1E-05); the most significantly changed category with longer tails in the mutants was major carbohydrate metabolism (p = 3.9E-08), followed by genes encoding proteins involved in amino-acid activation for translation (p = 1.9E-07) and proteins involved in cell division (p = 2.7E-07).

The poly(A) tail is known to stabilize transcripts, and shortening of the poly(A) tail often serves as a first step towards degradation of the mRNA by rendering the mRNA susceptible to decapping and 5’-3’ degradation, or to 3’-5’ degradation by the exosome [11,12,13,14]. Therefore, we predicted that there should be a correlation between the change in the long/short ratios and the change in overall transcript abundance between the wild-type and mutant samples; this prediction assumes that other parameters, such as the stability of the oligoadenylated form of a given transcript, do not differ between mutant and wild type. To estimate changes in overall transcript abundance between the genotypes, we combined the normalized read counts from the long- and short-tail fractions. Independent validation by qRT-PCR on biological replicate RNA samples to those used for the fractionation indicated that the above estimates robustly capture differences in transcript abundance between the *paps1-1* and wild-type samples (S2B Fig), as there was a significant correlation between fold-changes estimated by the two methods. The RNA-seq based estimates of the change in transcript abundance showed a highly significant positive correlation with the change in the long/short-ratio (Fig 2B; correlation coefficient 0.32; p-value <1.0E-50), which was even stronger for the 97 genes with significant tail-length change after multiple-testing correction (Fig 2B; correlation coefficient 0.4; p-value = 4.4E−05), suggesting that changing the poly(A)-tail length due to reduced PAPS1 activity alters transcript abundance. As described above, the steady-state poly(A)-tail length is not correlated with transcript abundance when comparing different transcripts in the same genotype (see above; S4A Fig), suggesting that other parameters are more important; by contrast, the correlation shown in Fig 2B supports the notion that a difference in the poly(A) tail of a given mRNA between different genotypes can affect the abundance of this transcript (see Discussion).

### Features differentiating PAPS1-sensitive and-insensitive transcripts

What differentiates transcripts whose poly(A)-tail length is sensitive to a reduction in PAPS1 function from the remaining ones? The changes in the long/short ratio showed no statistically significant correlation with the lengths of the annotated 3’ UTRs and only a very weak one with the number of annotated 3’ UTRs (S4D and S4E Fig). As observed for tail length in wild type, the extent of tail-length change between wild type and mutant was negatively correlated to transcript stability (Fig 2C). More stable transcripts also showed more pronounced shortening of the poly(A) tail in the mutant; however, this correlation was weaker than with tail length in wild type (Fig 2A). The base composition in the region 200 bp upstream and 200 bp downstream of the annotated 3’-cleavage sites did not differ between the 1000 genes with the lowest p-values for the change in the long/short ratio and 1000 randomly selected genes (S5A Fig). We used the 1000 genes with the lowest p-values from the test for a poly(A)-tail change between mutant and wild-type, even though most of them were not significant after correcting for multiple testing, as a comparison based only on the 97 significantly changed transcripts would have very limited sensitivity.

To determine whether there are sequence motifs enriched in the 3’ regions of those transcripts that show a high value for the change in the long/short ratio, we tested for all possible hexamer sequences whether genes with the hexamer present in the 100 bp before the polyadenylation site behave differently in the *paps1-1* mutant-vs-wild type comparison than genes without the hexamer. This analysis identified several hexamer motifs as significantly associated with an altered tail length in the mutants (S5 Table and S5C Fig); however, the effect sizes of these hexamers on poly(A)-tail change in *paps1* are small to minimal and/or the hexamers are rare (e.g. CGCCGA), indicating that they explain very little of the observed effects in the mutant. The hexamer AATAAA represents the canonical poly(A) signal in metazoans, and many variations of this hexamer are found upstream of plant cleavage sites [37]. We tested whether presence or absence of individual variations of this hexamer were associated with differential poly(A)-tail length in mutant and wild-type; while two variants (AAAAAA and AAGAAA) showed a statistically significant association with altered poly(A)-tail length, the effects were extremely weak (S5B and S5C Fig). Thus, a high transcript stability appears to contribute to a higher sensitivity of the transcript to reduced PAPS1 activity. However, neither our unbiased nor more targeted motif analysis identified simple sequence motifs with a strong individual effect on poly(A)-tail length in *paps1* mutants.

### Phenotypic effects of changes in poly(A)-tail lengths in *paps1* mutants

**Fig 3:**
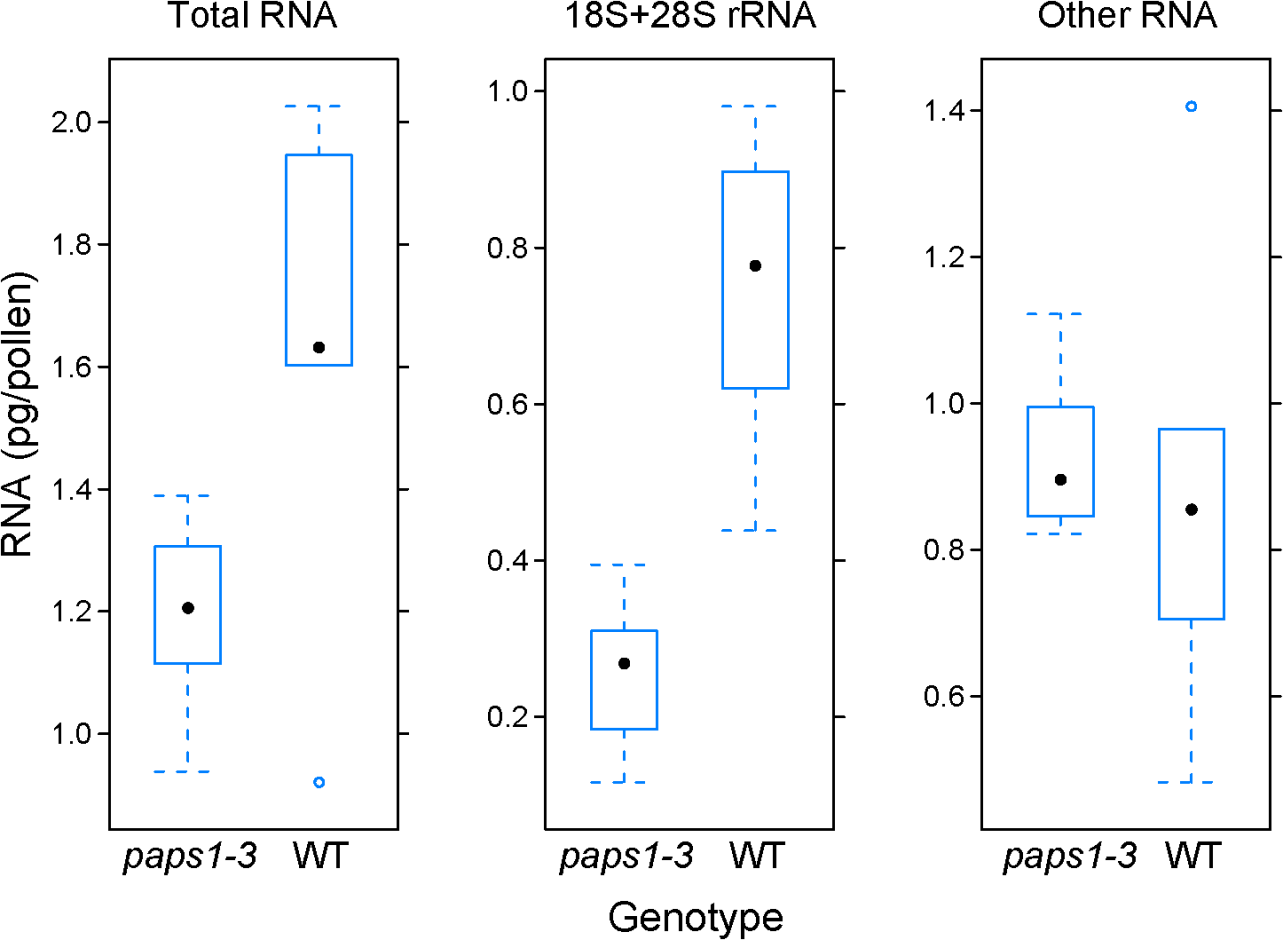
Role of *PAPS1* in ribosome biogenesis. Quantification of total RNA (left), combined 18S and 28S rRNA (middle), and remaining non-ribosomal RNA (right) in pg per pollen grain from *paps1-3* mutant and *PAPS1 pLat52*::*DsRED* wild-type pollen grains produced by *paps1-3*—*/ PAPS1 pLat52*::*DsRED* transheterozygous plants. Pollen grains were flow-sorted based on DsRED fluorescence and extracted total RNA was subjected to Bioanalyzer quantification. RNA amounts were related to the known number of pollen grains in the sorted fractions. Values represent five biological replicates; black dot is the median value, open blue circles show outliers as determined using the R/lattice boxplot.stats function.

To test whether the reduced poly(A)-tail lengths of transcripts encoding ribosomal proteins (see above) were functionally relevant, we sought to measure ribosome content in *paps1* mutants. As ribosomal RNA (rRNA) is rapidly degraded when not incorporated into functional ribosomes [40,41], the amount of ribosomal RNA can be used as a proxy for ribosome content. Quantifying the proportion of ribosomal RNA in total RNA samples by a BioAnalyzer RNA chip indicated that there was no significant change in the relative amount of ribosomal RNA in *paps1-1* mutants (S6A Fig). As the *paps1-1* mutant is not a null allele, we sought to determine whether the complete loss of *PAPS1* function in the *paps1-3* T-DNA insertion mutant would cause an effect on ribosome biogenesis [32]. As no *paps1-3* homozygous mutants can be obtained due to a male gametophytic defect, we used a fluorescence-marking system [42] to sort *paps1-3* mutant and wild-type pollen from heterozygous plants using fluorescence-activated cell sorting (FACS) [43]. A line carrying a pollen-expressed *pLat52*::*DsRED* reporter construct inserted about 290 kb from the wild-type *PAPS1* allele was crossed to *paps1-3* heterozygous plants, and *paps1-3*—*/ PAPS1 pLat52*::*DsRED* transheterozygous plants were selected. After meiosis, these plants produce roughly 50% *paps1-3* mutant pollen lacking DsRED fluorescence and roughly 50% *PAPS1* wild-type pollen with DsRED fluorescence (S6B Fig), as well as a negligible number of recombinant pollen grains. Isolation of total RNA from FACS-sorted pollen grains and Bioanalyzer analysis indicated that *paps1-3* mutant pollen grains contain a significantly reduced total RNA amount per pollen grain, which is due to a specific reduction in ribosomal RNAs, while non-rRNA was very similar in amount per pollen grain between the two genotypes (Fig 3). Thus, characterization of this null-mutant defect suggests that the shortened tails of transcripts encoding ribosomal proteins cause a reduced accumulation of these proteins and, as a consequence, of ribosomal RNAs in mature ribosomes.

If the set of transcripts with a change in the poly(A)-tail length indeed reflects the biological role of PAPS1, it should be possible to identify additional functions of PAPS1 by analysing this set of transcripts. To this end, we defined the set of 400 transcripts with the lowest p-values when comparing their long/short-ratios in *paps1* mutants versus wild type, and compared this list with lists of the 400 most strongly affected genes from each of 600 published microarray experiments using MASTA [44]. Amongst the 18 microarray experiments with the strongest overlap (representing the top 3%), there were three that modulated the redox status (S6 Table). In particular, these involved experiments with a line overexpressing the thylakoid-localized form of ascorbate peroxidase (tAPX), a scavenging enzyme for the reactive oxygen species H_2_O_2_ [45].

**Fig 4:**
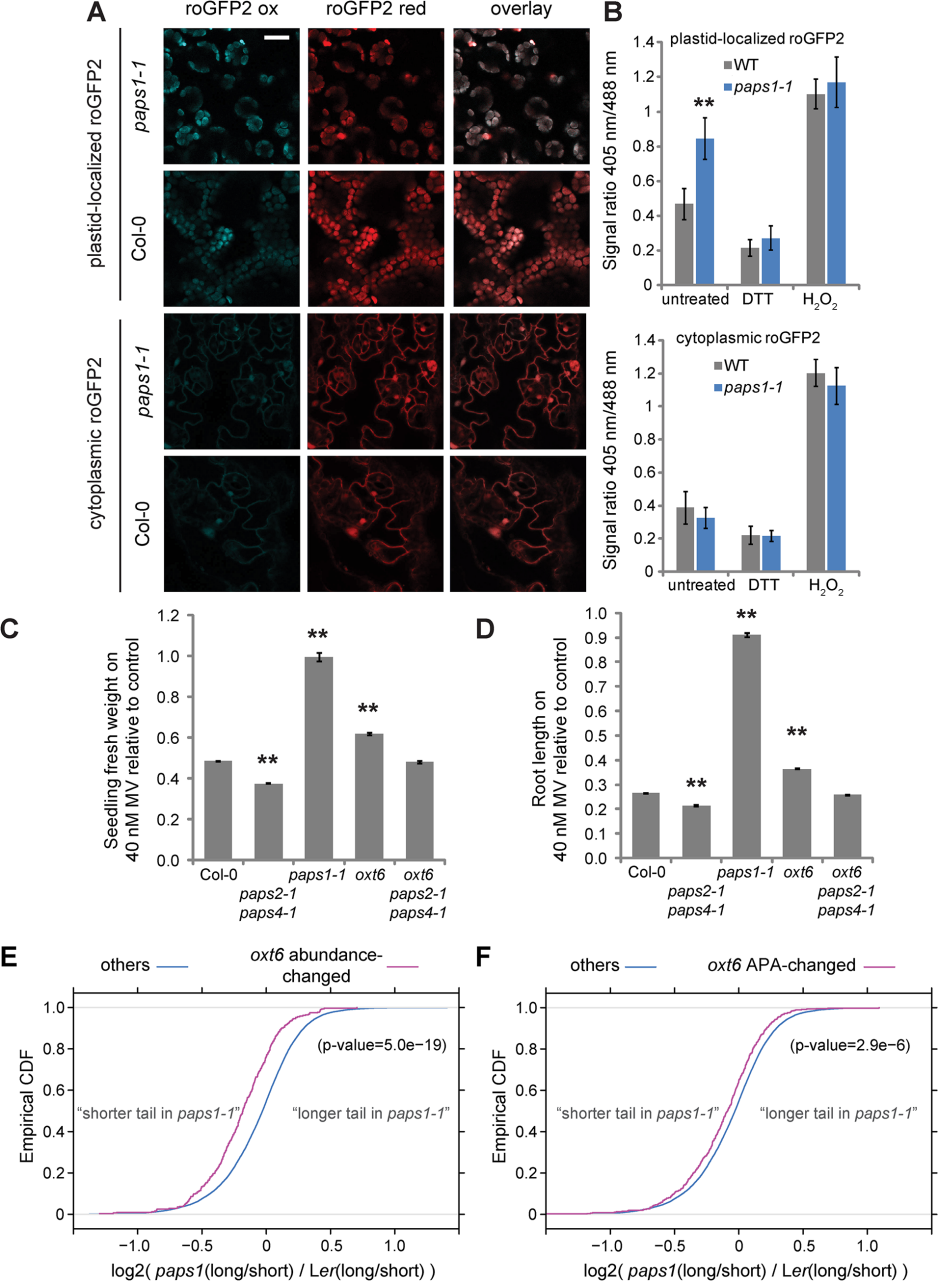
Role of *PAPS1* in redox homoeostasis and oxidative-stress response. (A) False-colored images showing roGFP2 fluorescence in the two genotypes and subcellular localizations indicated after excitation with 405 nm (roGFP2 ox) and 488 nm (roGFP2 red), and overlay of the two images. While cytoplasmic roGFP2 was imaged in the epidermis, plastid-localized roGFP2 was imaged in mesophyll cells, as in the epidermis it was only visible in stomatal guard cells. Scale bar is 20 μm. (B) Quantification of the ratio of roGFP2 fluorescence emission after excitation with 405 nm and 488 nm. DTT, H_2_O_2_: controls after treating plants with DTT to reduce and H_2_O_2_ to oxidize roGFP2 protein. Values are mean ± SEM of three biological replicates. (C) Fresh-weight analysis of seedlings of the indicated genotypes treated with 40 mM methyl-viologen relative to untreated controls. Values are mean ± SEM of at least 20 seedlings per genotype. (D) Root length of seedlings of the indicated genotypes treated with 40 mM methyl-viologen relative to untreated controls. Values are mean ± SEM of at least 20 seedlings per genotype. **: significantly different from control at p<0.01 based on Student’s t-test with Bonferroni correction. Absolute values for fresh weight and root length are shown in S7 Fig (E,F) Empirical cumulative distribution functions (CDF) of tail-length change in *paps1-1* mutants for transcripts with altered abundance (E) or altered alternative-polyadenylation (APA) patterns in *oxt6* mutants (F) versus genomic background (others). Indicated p-values are from a Wilcoxon rank-sum test.

Genes downregulated in *tAPX* overexpressing plants can be assumed to be induced by H_2_O_2_, while H_2_O_2_-repressed genes are expected to be upregulated in *tAPX* overexpressors. Of the 40 genes in the overlap between the most similar microarray and our experiment, 38 are more strongly expressed in *tAPX* overexpressors than in wild-type, while two show a lower abundance, and all of the 40 genes show evidence of a shorter poly(A) tail in *paps1* mutants (S7 Table). Assuming that at least some of these presumed H_2_O_2_-related genes with an altered poly(A)-tail length feed back on H_2_O_2_-levels and/or more generally the cellular redox status, we predicted a change in the cellular redox status in *paps1* mutants. To address this, we introgressed two reporter lines expressing a redox-sensitive form of GFP, localized either in the cytoplasm or in the chloroplasts, into the *paps1-1* mutant (Fig 4A) [46]. The emission maxima of this reporter depend on the excitation wavelength and the local redox potential. The reduced form emits most light at an excitation wavelength of 488 nm and the oxidized form at 405 nm. This makes it possible to use the ratio of fluorescence emission at 527 nm after excitation with the two wavelengths as a measure of the local redox potential. There was no difference in this ratio between mutant and wild-type leaves for the cytoplasmic reporter (Fig 4A and 4B). By contrast, the spectral ratio for the chloroplast-localized reporter was significantly altered in *paps1-1* mutant leaves, indicative of a more oxidizing environment in the mutant chloroplasts than in wild type (Fig 4A and 4B). To determine whether PAPS1 function not only influences the steady-state chloroplast redox potential, but also the physiological response to redox changes such as ROS accumulation, we treated plants with the herbicide paraquat (methyl viologen) that causes the increased production of superoxide and H_2_O_2_ in chloroplasts. Surprisingly, *paps1-1* mutant seedlings were significantly more resistant to paraquat treatment than wild type, both regarding their root and their shoot growth (Figs 4C, 4D, S7A and S7B). This effect is reminiscent of the protection against the effects of high-level ROS accumulation afforded by previous exposure to lower levels of ROS in tobacco plants [47]. By contrast, *paps2 paps4* double mutants were more sensitive to oxidative stress in both assays, suggesting opposite functions of PAPS1 and the other two isoforms in this process.

A link between 3’-end processing and oxidative-stress resistance has been documented before. Mutants lacking the activity of the CPSF30 subunit OXT6 are more resistant to oxidative stress than wild-type plants [48]. OXT6/CPSF30 influences the choice of 3’-cleavage and polyadenylation sites in a large number of *A*. *thaliana* genes, with an enrichment for stress-response and defense genes [36]. To determine whether the increased resistance to oxidative stress in *oxt6* and in *paps1* mutants was due to effects on the same set of genes, we determined the distribution of tail-length changes in *paps1* mutants relative to the transcriptome-wide background for transcripts with OXT6-dependent abundance changes or alternative polyadenylation (Fig 4E and 4F). The set of transcripts whose abundance is altered in *oxt6* mutants showed a significant shortening of the poly(A) tail in *paps1* mutants (Fig 4E), and the same was seen, albeit to a lesser extent, for the transcripts with OXT6-dependent alternative polyadenylation (Fig 4F). To test genetically for an interaction between *PAPS1* and *OXT6*, we sought to analyze the corresponding double mutant. No double homozygous plants could be isolated, and in the progeny of *oxt6/oxt6; paps1-1/PAPS1* plants, only a minority was heterozygous for the *paps1-1* mutation (Table 1). This suggests both synthetic lethality of the double mutant embryos and a functional defect in double mutant gametophytes, supporting the notion that both proteins act on a shared set of targets. By contrast, *oxt6 paps2 paps4* triple mutants were viable and showed an intermediate phenotype under oxidative stress treatment relative to *oxt6* single and *paps2 paps4* double mutants (Fig 4C and 4D).

**Table 1:** Distribution of *PAPS1* genotypes in the progeny of *paps1-1/+*; *oxt6/oxt6* plants.

	*PAPS1/PAPS1*	*PAPS1/paps1-1*	*paps1-1/paps1-1*
observed	111	67	0
expected	44.5	89	44.5
Chi-square	3.77351E-33		
expected for embryo lethality of double mutant	59	119	
Chi-square	1.23501E-16		

Progeny from *paps1-1/+; oxt6/oxt6* plants were genotyped for the *paps1-1* mutant and *PAPS1* wild-type allele (observed). Expected frequencies are given for Mendelian segregation assuming normal viability of the double mutant (expected) and for embryo lethality of the double mutant (expected for embryo lethality of double mutant). p-values for Chi-square tests against these two expectations are indicated.

Taken together, these observations suggest that as a result of the defective polyadenylation of a subset of transcripts *paps1-1* mutants accumulate more ROS in their chloroplasts, specifically more H_2_O_2_, and are at the same time more resistant to exogenously induced overaccumulation of ROS. While OXT6 and PAPS1 target overlapping sets of transcripts, PAPS2 and PAPS4 act independently of them, providing yet another example for functional specialization between the PAPS isoforms.

### Overlap of transcripts with an altered poly(A)-tail length and the PAPS1 co-expression module

**Fig 5:**
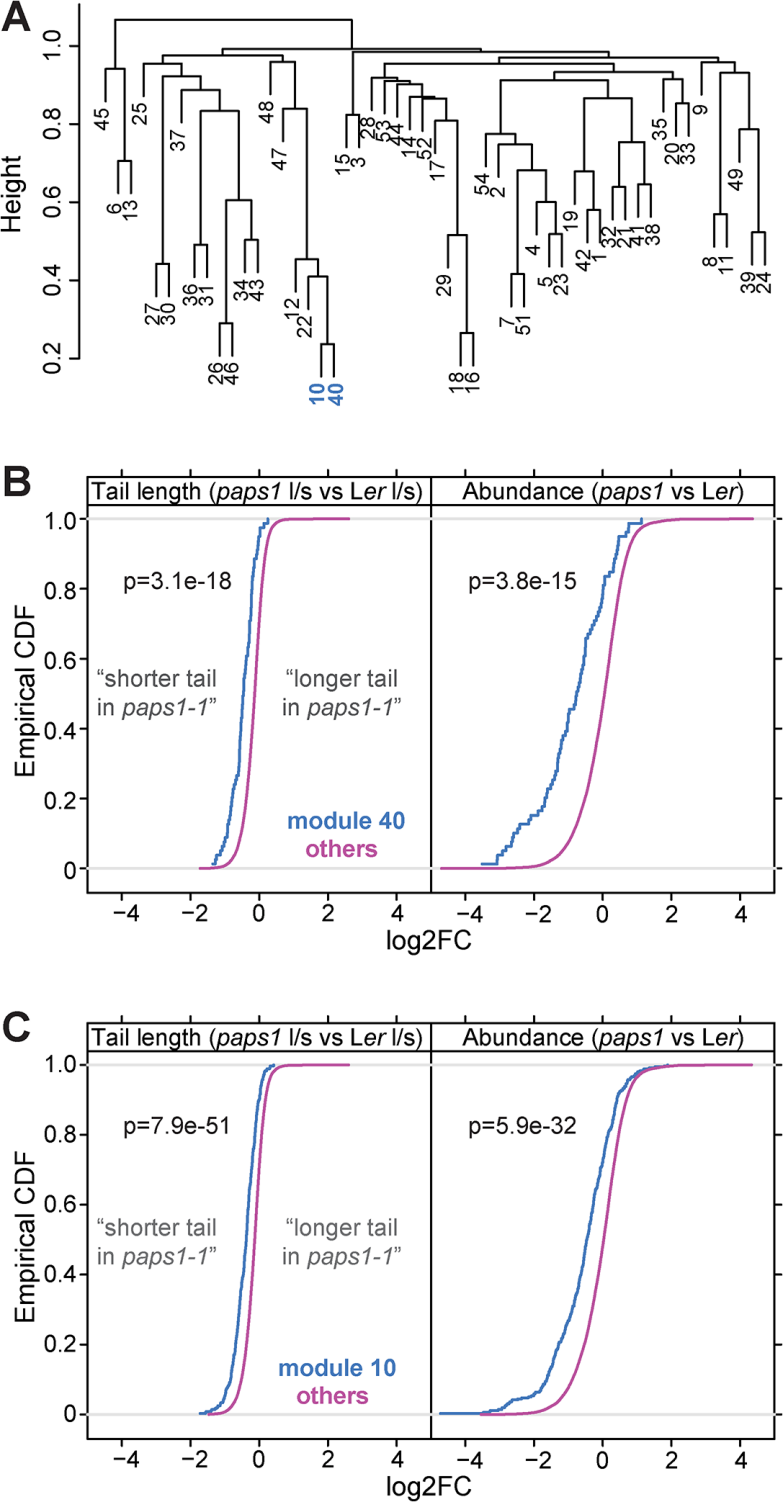
Genes coexpressed with *PAPS1* are strongly affected in *paps1* mutants. (A) Clustering analysis of coregulated modules across publicly available microarray data. *PAPS1* is part of module 10. Modules 10 and 40 are highlighted. (B,C) Empirical cumulative distribution functions (CDF) of tail-length change (left) and mRNA abundance change (right) in *paps1-1* versus wild type for genes in coregulated modules 10 (B) and 40 (C) compared to all other transcripts not in the respective module (other). p-values are from Wilcoxon rank-sum tests. Negative log2FC means shorter tail and reduced abundance in *paps1* mutants compared to wild type, respectively.

Our findings indicate that the *Arabidopsis thaliana* genome encodes poly(A)-polymerase isoforms with distinct functions that appear to reflect the preferential polyadenylation of sets of transcripts. This raises the possibility that the plant could exploit this specificity to modulate gene expression by altering the relative activities of the isoforms. If this were the case, a plausible prediction would be that alterations in PAPS1 activity in response to environmental or internal cues would co-ordinately affect PAPS1-targeted transcripts. We tested this prediction by exploiting the availability of a large number of *Arabidopsis* microarray experiments, under the assumption that altered *PAPS1* mRNA expression levels would be translated into corresponding changes at the protein level. Based on 14,115 publicly available microarray experiments, gene modules showing co-regulated expression across these experiments were identified using WGCNA [49] (S8 Table). *PAPS1* was part of a module (no. 10) comprising 564 genes; a second module (no. 40) comprising 104 genes was very similar to module 10 based on hierarchical clustering (Fig 5A). For each of the modules, the distribution of estimated poly(A)-tail length changes in *paps1* versus wild type was compared to the transcriptome-wide background. This analysis identified the two modules 10 and 40 as the ones with the highest differences in the median tail-length change (S9 Table and Fig 5B and 5C). We extended this analysis to include the estimated changes in overall transcript abundance between *paps1* and wild type. This yielded the analogous result: The same two co-expression modules showed the highest differences in the median of the distribution of abundance changes (S9 Table and Fig 5B and 5C). Thus, the genes that are co-expressed with *PAPS1* across available microarray experiments are the most strongly affected genes in *paps1* mutants regarding poly(A)-tail changes and total abundance.

## Discussion

Here, we have used a fractionation-based RNA-seq method to obtain a transcriptome-wide view of the mRNAs whose polyadenylation status changes in *paps1* mutants. This has identified two additional biological functions of PAPS1, shed light on the features that determine a transcript’s sensitivity to altered PAPS1 activity levels, and suggested that modulation of PAPS1 activity is used by the plant to coordinately influence the expression of a subset of transcripts.

Two broad classes of techniques have been described to determine poly(A)-tail length on a transcriptome-wide level. The first class fractionates cellular mRNA based on the length of the poly(A)-tail and then determines transcript abundances in the different fractions [16,50,51]; such techniques have for example been used to demonstrate modulation of poly(A)-tail length during yeast cell-cycle progression and circadian rhythms [10,50]. The second class uses next-generation sequencing to either directly sequence the poly(A) tail (TAIL-seq) [52], or incorporates biotinylated dUMP when complementing the poly(A)-tail of captured sequence tags on the Illumina instrument, then sequences the 3’ UTR next to the poly(A) tail and finally measures the fluorescence of fluorophore-tagged streptavidin bound to the biotinylated dUMP (PAL-seq) [17]. The fractionation-based techniques provide more indirect estimates of poly(A)-tail length than those from the second class, and the fractionation step appears to be rather sensitive to even slight fluctuations in conditions. Nevertheless, we find that our estimates of poly(A)-tail length in wild-type seedlings are in good agreement with measurements obtained by PAL-seq from mature leaves; also the RNA-seq based estimates of tail-length changes for *SAUR* mRNAs in *paps1* mutants are in agreement with our previous experimental results [32], indicating that our fractionation-based method provides reliable and biologically meaningful estimates of poly(A)-tail length. This notion is further supported by our identification of two additional biological functions for PAPS1 solely on the basis of our poly(A)-tail length analysis. Thus, we conclude that our analysis has meaningfully captured the transcriptome-wide effect of PAPS1 activity on mRNA poly(A)-tail lengths. As the *paps1* mutation affects the activity of a canonical nuclear poly(A) polymerase, we consider the observed changes in poly(A)-tail lengths in the mutant as a result of altered nuclear polyadenylation of the corresponding pre-mRNAs, rather than their selective deadenylation and degradation in the mutant. While selective changes in mRNA stability and increased deadenylation under heat stress have been described, for example for transcripts encoding ribosomal proteins and ribosome biogenesis factors [53], it appears unlikely that this effect explains the changes we observe, as both wild-type and *paps1-1* mutant plants were shifted to 28°C (if anything only a very mild heat stress for *Arabidopsis thaliana*; [54]) before harvesting, so any effects of this treatment should occur equally in both genotypes. Repeating the experiment with only nuclear RNA will be required to validate our above assumption.

Our analysis of poly(A)-tail length in wild type identifies transcripts encoding ribosomal subunit proteins as one of the classes with the shortest poly(A) tails. While the functional significance of this finding is currently unclear, the same effect has been observed in a previous study in yeast, *A*. *thaliana* leaves, *Drosophila*, zebrafish and human cell lines, suggesting a very broad conservation of this relationship [17]. Our analysis also identifies a negative correlation between a transcript’s half-life time and its steady-state poly(A)-tail length distribution. A similar trend has been reported in some studies of yeast and human cell lines, although the results within these organisms differ depending on the details of the half-life and poly(A)-tail measurements [17,50,52]. The main pathway of mRNA degradation occurs via de-adenylation at a transcript-specific rate followed by rapid exonucleolytic digestion of the resulting oligoadenylated form [1,12,14]. The observed transcriptome-wide negative correlation between half-life and poly(A)-tail length suggests that the stability of the oligoadenylated form is a more important determinant of mRNA half-life than the rate of de-adenylation in plants: When the rate of transcription and de-adenylation are the same, a higher stability of the oligoadenylated form would result in both a longer half-life and a shift in the distribution of poly(A) tails towards shorter lengths, mirroring the correlation we observe. By contrast, keeping the rate of transcription and the stability of the oligoadenylated form constant, but decreasing the rate of de-adenylation would result in a longer half-life, but at the same time a longer poly(A) tail on average.

While the steady-state poly(A)-tail length is negatively correlated with transcript stability (Fig 2B), there is only a weak relation between poly(A)-tail length and transcript abundance across different transcripts in the same genotype, suggesting that other parameters are more important in determining transcript abundance. However, comparing the same transcript between *paps1* mutants and wild type shows a positive correlation between the change in poly(A)-tail length and the change in transcript abundance (Fig 2C). This is consistent with the notion that, all else being the same, PAPS1-target transcripts that are appended with a shorter poly(A) tail in the *paps1* mutant require less time to be deadenylated, resulting in faster turn-over and reduced transcript abundance compared to wild type.

A puzzling and counterintuitive observation is that some transcripts appear to have a longer poly(A) tail in the *paps1* mutant than in wild type. With PAPS1 activity strongly reduced, these longer poly(A) tails should reflect the activity of the other two isoforms PAPS2 and PAPS4. It is thus conceivable that for some transcripts PAPS1 competes with PAPS2/4 for polyadenylation in wild type, and that for unknown reasons polyadenylation by PAPS1 results in a shorter steady-state distribution of poly(A) tails on these transcripts. Comparing the results presented here with those from an analogous analysis of *paps2 paps4* double mutants will be required to test this possibility further.

While a transcript’s sensitivity to reduced PAPS1 activity was related to its stability and number of alternative polyadenylation sites, we did not identify any simple sequence feature, i.e. hexamer motif, that would robustly discriminate PAPS1-sensitive and insensitive transcripts. Several explanations are conceivable for this, such as combinatorial action of partly redundant sequence elements or a greater importance of secondary structure than nucleotide sequence in the 3’ UTR, and further experimentation will be required to discriminate between these.

A link between 3’-end processing and oxidative-stress resistance has been documented before by studying the function of the CPSF30 subunit OXT6 [36,48]. Both the sets of transcripts whose abundance is changed in *oxt6* mutants and those with evidence of OXT6-dependent alternative polyadenylation had a significantly shorter poly(A)-tail distribution in *paps1* mutants versus wild type. Also, no *oxt6 paps1-1* double mutants could be identified due to synthetic lethality and a defect of double mutant gametophytes. While this indicates a functional interaction between the two factors, likely via their action on an overlapping set of transcripts, no binding of PAPS1 to OXT6/CPSF30 could be detected in comprehensive yeast two-hybrid interaction tests [29]. Unraveling the mechanistic basis of the observed overlaps may provide insight into the way that PAPS1 is recruited preferentially to some transcripts over the PAPS2/4 isoforms and vice versa.

Just like the other phenotypes of *paps1* mutants [31,32], their higher resistance to ROS and synthetic lethality with *oxt6* are specific effects of changes in PAPS1 activity, and are not seen in mutants lacking the activity of the other two canonical nuclear poly(A) polymerases PAPS2 and PAPS4. These phenotypes in *paps1* mutants correlate with altered poly(A)-tail length of mRNAs whose abundance changes in plants with an altered redox status in chloroplasts. What is unclear at present is whether the changes in expression of these transcripts in response to redox alterations are caused–at least partly–by dynamic changes in their poly(A)-tail length, mediated by altered activities of the PAPS1 isoform. Answering this question might identify a concrete example where the balance of enzymatic activities of the different nuclear PAPS isoforms is altered in response to environmental factors in order to coordinately modulate the expression levels of distinct subsets of genes. While such an example is still missing, our co-regulation analysis provides strong support that this mechanism is being used by the plant. Searching for modules of co-expressed genes across more than 14,000 published *A*. *thaliana* microarrays identified two modules of 564 and 104 genes showing close co-expression with *PAPS1*. Strikingly, the genes in these modules behave very differently from the rest of the genome in our poly(A)-tail profiling of *paps1* mutants relative to wild-type. Genes in the two modules show the strongest shortening of the poly(A)-tail in the *paps1* mutants compared to all other identified co-expression modules. An analogous result was seen when considering the estimates of overall abundance changes in *paps1* mutants versus wild type. These highly statistically significant results were obtained by comparing independent large-scale data sets generated by independent methods. In our view, this provides strong evidence that the co-expression seen in the microarray experiments is at least partly due to changes in PAPS1 activity in response to environmental or internal cues, presumably via its effects on the polyadenylation of its preferential target mRNAs. Thus, our analysis supports the existence of an additional layer of gene regulation based on functional specialization amongst canonical nuclear PAPS isoforms.

In summary, we demonstrate here that a fractionation approach based on poly(A)-tail length coupled with RNA-seq can be used to identify groups of target transcripts that are preferentially polyadenylated by a particular PAPS isoform. Analysis of these groups uncovered two novel roles for the PAPS1 isoform from *A*. *thaliana* in ribosome biogenesis and in redox homeostasis and oxidative-stress resistance. Data integration with publicly available microarray experiments provides strong evidence that the relative activities of the different PAPS isoforms are modulated in plants to co-ordinately alter the expression of groups of transcripts.

## Materials and Methods

### Plant materials and growth conditions

The *paps1-1* mutant, the *paps1-1* mutant introgressed into Col-0, the *paps2-1 paps4-1* mutant, the *oxt6* mutant and the *roGFP*-expressing lines have been described before [32,46,48]. The mapped *pLat52*::*XFP* insertion lines have been described before [42]. The line used here, 1376, is located at position 6,488,210 on chromosome 1, approximately 290 kb from the *PAPS1* locus. Growth conditions were as described before [32]. To ensure comparability of the developmental stage despite the slower development of *paps1* mutants, *paps1* mutant seeds were germinated two days before the other genotypes.

### Generation of spike-in control RNAs

#### 30-A control RNA

Plasmid pJill_SV1 (35S::omega Leader::YFP in pBlueML) was linearized with BsrGI (NEB) and gel-purified. An equimolar mixture of GTO277 and GTO278 was heated to 95°C in a heating block, allowed to slowly cool to room temperature, and ligated to the linearized pJill_SV1, resulting in plasmid pGT2c, with the orientation of the insert confirmed by sequencing. pGT2c was digested with NcoI to remove the omega leader and religated, resulting in plasmid pGT2d. pGT2d was linearized with BamHI, phenol:chloroform purified, and used as template for *in-vitro* transcription.

#### 75-A control RNA

Plasmid pJill_SV1 (35S::omega Leader::YFP in pBlueML) was linearized with KpnI (NEB) and gel-purified. An equimolar mixture of GTO289 and GTO290 was heated to 95°C in a heating block, allowed to slowly cool to room temperature, and ligated to the linearized pJill_SV1, resulting in plasmid pGT3b, with the orientation of the insert confirmed by sequencing. During this process, we also identified a clone that had the insert twice with a total of 134 A, which was termed pGT3a. pGT3b was linearized with XmaI, phenol:chloroform purified and used as template for *in-vitro* transcription.

#### 134-A control RNA

Plasmid ML1004 (AlcA::omega Leader in pBlueML) was digested with SalI and XhoI to remove the omega leader and religated. The resulting plasmid was termed pGT4. The 134-A fragment from plasmid pGT3a was isolated by digesting with KpnI, gel-purified and ligated into the KpnI linearized pGT4, resulting in clone pGT5, with the orientation of the insert confirmed by sequencing. pGT5 was linearized with BamHI, phenol:chloroform purified, and used as template for *in-vitro* transcription.

Oligonucleotide sequences are given in Table 2.

**Table 2:** Oligonucleotides used for generating spike-in control RNA templates.

Name	Sequence
GTO277	GTACATTTTTTTTTTTTTTTTTTTTTTTTTTTTT
GTO278	GTACAAAAAAAAAAAAAAAAAAAAAAAAAAAAAA
GTO289	CAAAAAAAAAAAAAAAAAAAAAAAAAAAAAAAAAAAAAAAAAAAAAAAAAAAAAAAAAAAAAAAAAAAAAAAAAAAGGTAC
GTO290	CTTTTTTTTTTTTTTTTTTTTTTTTTTTTTTTTTTTTTTTTTTTTTTTTTTTTTTTTTTTTTTTTTTTTTTTTTTTGGTAC

Sequences are given for oligonucleotides used to generate the templates for *in vitro* transcription of spike-in control RNAs.

### In-vitro transcription

One microgram of each linearized plasmid was diluted in 13.6 μl of H_2_O. Subsequently 2 μl 5 mM NTP solution (BioLine), 2 μl 10x T7 transcription buffer (NEB) and 1 μl 2 mg/ml BSA (NEB) were added. After mixing, 1 μl RNase Inhibitor (Promega) and 0.4 μl T7 Polymerase (NEB) were added. After incubating for 2 hours at 37°C, the reactions were stopped by adding 1.5 μl TURBO DNase (Ambion) and incubating for 30 min at 37°C. Phenol:Chloroform extraction on the samples was carried out. RNA concentrations were measured with the Picodrop and a mix of all RNAs with a concentration of 1 ng/μl was made. 1 μl of this mix was added to each RNA sample before fractionation (see below).

### Fractionation based on poly(A)-tail length and RNA-seq

The mRNA fractionation was carried out with the Promega PolyATract System 1000 and the protocol modified as follows: The GTC, DIL, ß-mercaptoethanol (BME), biotinylated oligod(T), 0.5x SSC and H_2_O were allowed to reach room temperature. Forty-one microliter of BME were added per ml of GTC (GTC/BME) and 20.5 μl BME were added per ml of DIL and preheated to 70°C. The SSC buffer was diluted to a concentration of 0.085x. In a 2 ml tube, 80 μg of total RNA (in a maximum of 40 μl) were mixed with 400 μl GTC/BME, 15 μl biotinylated oligo d(T) (Promega) and 816 μl DIL/BME and heated to 70°C for 5 min. Afterwards the samples were spun at 13 000 rpm for 10 minutes at room temperature. In the meantime the paramagnetic beads were washed. Afterwards the beads were resuspended in 600 μl 0.5x SSC. The supernatant of the spun samples was added to the washed beads and the biotinylated oligod(T) was allowed to bind the beads by rotation at room temperature for 15 min. In the following step, the beads were captured and the supernatant transferred to a new tube (unbound fraction). The beads were washed three times with rotation for at least 5 min between each wash step. Afterwards, the beads were resuspended in 400 μl of 0.085 x SSC and rotated for 10 min at room temperature. The beads were captured and the eluate transferred to a new tube (short fraction). This step was repeated once (total of 800 μL). The beads were then washed with 400 μl nuclease free water (rotation for 10 min) twice and the eluates transferred to a new tube (800 μl, long fraction). All collected samples where centrifuged for 10 min at 13000 rpm, 4°C to remove any transferred beads. Then 0.1 vol of Co-precipitant Pink buffer (BioLine) were added and the samples mixed well. Afterwards 30 μg of Co-precipitant Pink (BioLine) were added, mixed well, and 1 vol 100% ethanol was added. The samples were incubated overnight (15–16 hours) at -20°C. In the next step, the samples were centrifuged at 13000 rpm, 4°C for 30 min. The supernatant was removed, the pellet washed with 500 μl 80% ethanol, dried, and dissolved in 15 μl DEPC treated water.

1 μl of each fraction was taken for reverse transcription, the rest was used for Illumina sequencing using the TruSeq protocol with a read length of 50 bp and single-end protocol. The eight long and the eight short fractions were each sequenced on one lane of a HiSeq2000 instrument. Sequencing was performed at LGC Genomics (Berlin, Germany).

### Analysis of bulk poly(A)-tail lengths

Bulk poly(A)-tail lengths of mRNAs in the short and long fractions were determined as described before [32].

### qRT-PCR validation

1 μl of each sample from the oligo(dT) fractionated mRNA was used for reverse transcription with oligo(dT)17 primers using SuperScript III. Expression levels were analysed using a Roche LightCycler 480.

For estimating changes in total transcript abundance, RNA was extracted from *paps1-1* mutant and L*er* wild-type seedlings grown and treated in parallel to those used from RNA fractionation. Reverse-transcription and qRT-PCR were performed as above using primers listed in Table 3 and Table 4.

**Table 3:** Primers used for qRT-PCR against spike-in control RNAs.

Gene		Sequence
34A control	fw	CCGACAACCACTACCTGAGC
	rev	TCCATGCCGAGAGTGATCC
75A control	fw	AGACGTTCCAACCACGTCTT
	rev	GAAGGATAGTGGGATTGTGCG
134A control	fw	CTTCGGGATAGTTCCGACCT
	rev	CAGTACCAGAAAGTGCTCCGT

The indicated primers were used to determine levels of the spike-in control RNAs using qRT-PCR.

**Table 4:** Primers used for qRT-PCR against endogenous transcripts.

GTO420	AT1G80745_TFIIIC_qF	AGGCACATTGACATTCCAGCAG
GTO421	AT1G80745_TFIIIC_qR	CAAGGCATGTACCAATCGTTGC
GTO424	AT1G72930_TIR_qF	TCCGGCGATTGTTCAGGTGATG
GTO425	AT1G72930_TIR_qR	ATGCGAGTCCTTATGGGCCTTC
GTO426	AT2G31081_CLE4_qF	GGAAGAGAGCCCTTCAGATTCAGG
GTO427	AT2G31081_CLE4_qR	AGAACCGTTTGGCCGTCTTTCG
GTO428	AT5G67300_MYB44_qF	GAAGCGTGTGGGACAAGTAAG
GTO429	AT5G67300_MYB44_qR	GACGTTGGAGTGGGCTATG
GTO430	AT5G05200_aaarFKinase_qF	ACCGCGCTTAATGTGTGGTTTG
GTO431	AT5G05200_aaarFKinase_qR	AGAAACCCAATCCGGCCATCAC
GTO432	AT1G22240_Pumilio8_qF	TGCGCGAACTCATCTCTGTTCC
GTO433	AT1G22240_Pumilio8_qR	TGTGGCATGAAGTGAACCCTTGG
GTO438	AT4G37930_SMeth_qF	ACTATGCCCGCATCAGAAAGGTC
GTO439	AT4G37930_SMeth_qR	AGCATAGTCGAACGGTGAAGGG
GTO440	AT2G41100_TCH3_qF	TTCGACAAGAATGGTGATGGTTCC
GTO441	AT2G41100_TCH3_qR	TCCGCTTCGTTCATCAAGTCCTG
GTO442	AT2G24600_Ankyrin_qF	TGGAAAGGGAAATCGCTTGTAGGG
GTO443	AT2G24600_Ankyrin_qR	TGCGGTATGGTCACCAAAGATGC
GTO444	AT1G27410_Metal_qF	TCTAGTCGCTGTTCAATTGGTTGC
GTO445	AT1G27410_Metal_qR	CGGGAAACATGAACAAGGAGGTC
GTO446	AT5G59310_LTP1_qF	AGTGTTCATCGTTGCATCAGTGG
GTO447	AT5G59310_LTP1_qR	AGACATGGACTCAAGCTACTTGCC
GTO450	AT2G37870_LTP2_qF	TCGGCGTTCCTAAACGCTGTAAC
GTO451	AT2G37870_LTP2_qR	TGTAACGTCCACATCGCTTGCC
GTO452	AT1G06670_DEIH_qF	TTGCACCCAACCACGTCGTATC
GTO453	AT1G06670_DEIH_qR	TTCACTTTGCAGCCGAACCTTG
GTO454	AT2G42640_MAPKKK_qF	ATCCAAGCCCTGGAAAGGAGTC
GTO455	AT2G42640_MAPKKK_qR	TCTGGTTCCGACCAACAATCTGC
GTO456	AT3G15356_LegLec_qF	CACTCCTCGCACCAAACCTAATTC
GTO457	AT3G15356_LegLec_qR	TATCAGCGGCTGGGACAATGAC

The indicated primers were used to determine levels of endogenous transcripts using qRT-PCR.

### Extension poly(A)-tail length assay

Total RNA was extracted from nine-day old L*er* and eleven-day old *paps1-1* mutant seedlings after incubation at 28°C for two hours. RNA was extracted using Trizol and DNase-digested using Turbo DNase (Ambion). The extension poly(A)-tail length (ePAT) assay was performed as described [35]. To estimate the length of the transcript without the poly(A) tail, an aliquot of RNA was reverse-transcribed using the oligonucleotide 5’- GCGAGCTCCGCGGCCGCG(T)12VN-3’ to prime the reverse transcriptase; the sequence preceding the twelve T residues corresponds to the universal reverse primer used for the ePAT assay. For single PCR reactions, 34 cycles were used; for nested-PCR assays, 24 cycles were used for the first PCR, of which 1 μl was used as template for the nested PCR (20 μl reaction) with 24 cycles. PCR-products were analyzed on an Agilent Bioanalyzer 2100 using the DNA 1000 chip. Band intensities were extracted and analyzed as described [32]. To calculate the difference in the relative abundance between the ePAT and the (dT)12VN control products as a proxy for the poly(A)-tail length distribution, individual samples were normalized by dividing each value by the mean of all sample points within the region of interest. These relative abundances were averaged by sample type to finally subtract values for control products from ePAT products. We note that this is likely to underestimate the true poly(A)-tail length, as any PCR-products resulting from mispriming of the (dT)12VN control primer inside the poly(A) tail during reverse transcription will substract from the signal ascribed to the poly(A) tail.

The selection of candidate genes for ePAT was based on an initial analysis of the RNA-seq data. 40 candidates were chosen manually within the 250 genes with the lowest p-value for a tail-length change, requiring above-average expression as determined by a logCPM value of close to 4 or above. Of these 40 candidates, we were able to successfully establish primers for specific amplification of the 3’ UTR and poly(A) tail for 15 genes. Their analysis is presented in S3 Fig. The relatively low rate of success reflects the low sequence complexity and high AT-content of plant 3’ UTRs, in which primers have to be designed for ePAT analysis, in order to keep amplicon lengths short. Oligonucleotides used to assay the 15 transcripts are listed in Table 5.

**Table 5:** Primers used for ePAT assay.

AGI code	Single PCR primer	Nested PCR primer
AT5G48490	TGTGGCTACAAAAACTCTCCGTGGCTCGG	
AT5G11740	GCAGTCGCGGCTCTTGTTTTTGG	TCGTGTGTATTCCGAAGCAG
AT1G51400	GACGATGAGCCAAAGAGAGG	ATGCCTACCGCCAAGATATG
AT3G09390	GCAAGTGTGATCCTTGCACCTGC	AGCAACTCTGCCATGTGATG
AT5G61590	AGATCCGGCAAAGAAAGGAT	GGAGGAGAACCGATGTACCA
AT5G64140	CAAACTCGATTTCGCCTAGC	ACTGGTTCTCGTGGTCAGGT
AT5G48485	CATGAGCCAGGATGAGTTGA	TTCTGCTCTCCCCAAACAGT
AT5G18600	TTCATTGGTGGAGAGTTGGTC	GGTGCATTGTGGGTTTGACT
AT5G22880	TTGTGTTGCCTGGAGAGTTG	TGTGGTTTTGTAATGGAGGAGA
AT2G34585	CGGAGGCATCTGAGAAAGAG	CTTGTAAGCCTTGCCTGGAG
AT5G55290	CGTCGTTGGGATAATTGCTT	GGTCATTACTGCGACGGTCT
AT1G48440	GGTGGCCGTCGAAGTAGTAA	GCGCTGCAGGAATTCTACTC
AT4G31560	TCGCCATTCCTGCTACTCTT	CAAAATCGCTGACAAACTCG
AT1G01470	CTCAAAGACGTGAACCGTGA	TCGACCTTCCTGTTGTAGGG
AT3G44010	TCGCCGTTTGTTATTCCTCT	

The indicated primers were used to determine poly(A)-tail lengths of the indicated transcripts using the ePAT-test. Where nested PCR was used, primers for the first (“Single PCR primer”) and second (“Nested PCR primer”) rounds of PCR are indicated.

### Bioinformatic analysis

All the following analyses were done using R (R Core Team, 2015). Illustrations were done using the R package Lattice [55].

#### Preprocessing and read mapping

Barcoded reads were separated, adapter clipped and poly(A)-filtered. Ribosomal RNA reads were filtered using RiboPicker [56]. RNA-seq reads for all samples were mapped against the TAIR10 reference genome using TopHat2 [57] with a reference annotation from TAIR10. Output files were further processed using samtools [58] before counting reads using htseq-count [59] and quantifying gene expression using cufflinks [60] to get fpkm-values (fragments per kilobase of exon per million fragments mapped).

#### Estimates of poly(A)-tail length

Poly(A)-tail lengths were estimated by taking the relative abundance of a given gene in the long fraction compared to the short fraction. Differences between the *paps1* mutant and wild-type were addressed by comparing the long to short fraction ratios. Both calculations were done using the R/Bioconductor [61] package edgeR version 3.6,8 [62] with htseq-counts. Genes with 5 or less reads in one or more sequencing sample were filtered out. P-values were corrected for multiple testing using the Benjamini and Hochberg method [63]. Read counts were normalized using the TMM method [64].

#### Estimates of overall transcript abundance

Overall transcript abundance differences between *paps1* mutants and wild-type were approximated by comparing the combined relative abundances of long and short fractions between the two genotypes. Calculations were done using edgeR with htseq-count counts as described above. For comparison with the experimentally determined fold-changes based on qRT-PCR on replicate RNA samples, fpkm values were used to be able to also include lowly expressed genes.

#### Correlations of poly(A)-tail length with transcript features

Log2(poly(A)-tail length) estimates were correlated with mean seedling expression for public data sets obtained from NCBI SRA (SRR070570-71, SRR345562, SRR349697, SRR501594-97). Log2(poly(A)-tail length) differences between *paps1* mutant and wild-type were correlated with log2 estimates of overall transcript abundance differences. Both were correlated to maximum stop to cleavage site distance (termed 3' UTR lengths), number of 3' UTRs and half life in hours determined by [38]. 3' UTR information annotations were taken from TAIR10 and complemented with additional annotations from two recent large-scale sequencing studies [36,37]. Correlations and p-values were calculated using the R function cor.test.

#### Identification of overrepresented sequence motifs and sequence features

Hexamer sequence motifs were extracted for the last 100 bp of all annotated 3' UTRs. Significant motifs were identified using a Wilcoxon Rank-Sum test to compare poly(A)-tail length differences between *paps1* mutant and wild-type for genes with and without the motif at the end of its 3' UTR. Overall sequence compositions were described by plotting the relative nucleotide abundances 100 bp before and after the most distal predicted cleavage site.

#### MapMan and gene-family analysis

Significantly affected gene categories as defined in the MapMan Ontology [65] were identified using a Wilcoxon rank-sum test.

#### Construction and analysis of co-expression modules

Co-expression modules were generated using WGCNA [49] with 14,115 unique *Arabidopsis* ATH1 microarray data sets (S10 Table) from GEO [66], ArrayExpress [67] and NASCArrays [68]. Due to duplicated entries in one or multiple databases unique microarray data sets were identified using md5sum and then MAS5 normalized using the R/Bioconductor package affy [69]. The significance of difference in poly(A)-tail length and total abundance changes between genes within the modules compared to all other genes were calculated using a Wilcoxon rank-sum test. The differences in poly(A)-tail lengths and abundance estimations between the *paps1* mutant and wild type for genes in the PAPS1 containing module (numbered 10) as well as the most similar module (numbered 40) compared to all other genes were further analyzed. Changes were illustrated using an empirical cumulative distribution function plot.

### Estimates of ribosome content

To estimate the fraction of rRNA from total RNA, total RNA was extracted from 13 (*paps1* mutants) and 11 (other genotypes) days-old seedlings of the different genotypes grown at 28°C to obtain a severe *paps1-1* mutant phenotype [32]. Total RNA was separated on an Agilent Bioanalyzer using RNA chips according to the manufacturer’s protocol.

To determine ribosome content in *paps1-3* mutant pollen, pollen was collected from *paps1-3*—*/ PAPS1 pLat52*::*DsRED* plants. Plant growth and pollen isolation were performed as previously described [43]. Subsequently the pollen pellet obtained was resuspended in 2 ml of sperm extraction buffer and subjected to FACS.

Fluorescence-Activated Cell Sorting was carried out on a MoFlo high speed cell sorter (Beckman Coulter, Fort Collins, USA) equipped with a 488 nm laser (200 mW air-cooled Sapphire, Coherent) at 140 mW used for scatter and autofluorescence measurements. A 561 nm laser (50 mW DPSS, CrystaLaser) at 30 mW was used for DsRED excitation. Autofluorescence and DsRED were detected using 530/40 nm and 630/75 nm bandpass filters, respectively. The instrument was run at a constant pressure of 207 kPa (30 psi) with a 100 μm nozzle and frequency of drop formation of approximately 40 kHz. Cells were collected into RNA extraction buffer maintained at 4°C.

Total RNA was isolated using a RNeasy Mini kit (Qiagen) and RNA integrity and amount of ribosomal RNA was assessed using an Agilent 2100 Bioanalyser with RNA 6000 Nano Assay (Agilent Technologies).

### Measurements of redox-status and methyl-viologen treatment

#### Confocal imaging of roGFP

All pictures were taken with a LSM 710, AxioObserver (Zeiss) with the C-Apochromat 40x/1.20 W Korr M27 objective in multi-track mode with line switching between 405 nm and 488 nm excitation and constant emission acquisition at 527 nm wavelength. Settings were adjusted to untreated roGFP2 in the C*ol-0* background for cytoplasm and plastids separately and not changed for image acquisition of treated control or *paps1-1* mutant leaves in the respective compartment. Two pictures each from three different plants were taken.

The plant material was 11 day old *paps1-1; roGFP2* seedlings and 9 day old *roGFP2* seedlings grown on ½ MS plates under standard growth conditions. DTT and H_2_O_2_ controls were prepared as described [46].

Images were evaluated with the Carl Zeiss ZEN software in the following way: three different parts of each picture were selected and the values for the mean intensity for both and standard deviation channels were noted, then the ratio of oxidized/reduced mean intensity was calculated and averaged.

#### Methyl-viologen treatment

Seeds were sown out and stratified at 4°C for 4 days. Since the *paps1-1* mutant germinates late, the *paps1-1* containing plates were transferred to long day conditions two days before the other plates. After growth at 21°C during the day and 16°C at night under long day conditions, five day old seedlings were transferred from MS medium onto horizontal cut MS medium supplemented with 1% saccharose containing 40 nm MV or no MV. The MV was added to the medium just before pouring the plates. After 14 days, the individual fresh weight was determined and fotos of the plates were used to evaluate the individual root length of each plant using the program ImageJ. Five replicates consisting of at least five individuals were measured per genotype.

### Accession Numbers

RNA-seq data sets have been deposited in NCBI GEO under accession number GSE57690 (http://www.ncbi.nlm.nih.gov/geo/query/acc.cgi?acc=GSE57690).

## Supporting Information

S1 Fig Validation of fractionation. (A) Bulk poly(A)-tail analysis of the input and the unbound, short-tail and long-tail fractions from two biological replicates of wild-type seedlings. RNA was treated as described in the legend to Fig 1. Fragment sizes in nucleotides are indicated on the left based on a labeled RNA-size marker. The discontinuity at the top of the four right-most lanes is due to a tear in the gel. (B) RT-PCR products representing the three spike-in controls from the indicated fractions were separared by agarose gel electrophoresis. (C) Abundance estimates of the three spike-in control RNAs from qRT-PCR of the short- and long-tail fractions. Box-plots show the distribution of values from four biological replicates each. Filled black dots indicate the median value; open blue circles are outliers as determined using the R/lattice boxplot.stats function. (D) Clustering of samples based on fpkm ratios from the long-tail and the short-tail fractions (long/short ratio) after filtering out lowly expressed genes (fpkm < 0.5) and using 1 –correlation between each two samples as distance. Biological replicates cluster together well, when not considering lowly expressed genes subject to higher measurement variability using RNA-seq.
(PDF)

S2 Fig Validation of RNA-seq. (A) Correlation plots of abundance estimates from qPCR with those from RNA-seq for 15 transcripts from the indicated fractions. Pearson correlation coefficients, p-values and the number of included transcripts are shown in each panel. If fewer than 15 transcripts were included, this was because qPCR detection was unreliable. Asterisk indicates significant correlation at p<0.05. (B) Correlation of estimated fold-changes in total transcript abundance derived from qRT-PCR or from RNA-seq for the 15 transcripts from (A). qRT-PCR was performed on three biological-replicate RNA samples from seedlings grown in parallel to those used for the RNA fractionation. For the RNA-seq based estimates fpkm values were used to also include lowly expressed genes. The 15th gene excluded from the figure has no mapped sequencing reads in any of the three Ler samples; consistently, qRT-PCR also indicates a much higher abundance in *paps1-1* (log2FC > 10).
(PDF)

S3 Fig Validation of predicted poly(A)-tail length changes in *paps1-1* mutants. (A) Bioanalyzer results of ePAT (left) and (dT)_12_VN control (right) PCR products for *At3g09390* mRNA from *paps1-1* and L*er* wild-type samples. Three biological replicates were used per genotype. Size standard is shown in bp. (B) Analysis of the results from (A). Normalized signal intensity (averaged over the three biological replicates) is shown for the ePAT and control products from both genotypes (left) and for ePAT only (middle). Right panel shows the difference in relative signal intensities between the ePAT and control products. The broad shoulder to the right of the dip at 315 bp represents the poly(A) tail. The shift of the wild-type curve (red) towards longer products relative to the mutant curve (blue) indicates the longer poly(A) tail in the wild-type samples. (C) Differences of relative signal intensities between ePAT and control samples as determined in the right panel of (B) are shown for 15 transcripts. Numbers in the panels indicate the predicted log2-fold change of the poly(A)-tail length between *paps1-1* and wild-type samples based on the fractionation/RNA-seq approach. Green font indicates genes for which the ePAT assay indicates longer poly(A) tails from wild-type than from mutant samples (i.e. higher values for the purple than the blue curve at longer product lengths); black font indicates transcripts without a robust change, and red font denotes shorter poly(A) tails in wild-type than mutant samples.
(PDF)

S4 Fig Test for correlations between transcript features and poly(A)-tail length. (A-C) Correlation of transcript abundance (A), number of annotated 3’ UTRs (B) and length of the 3’ UTR (C) with estimated poly(A)-tail length in wild-type. (D,E) Correlation of number of annotated 3’ UTRs (D) and length of the 3’ UTR (E) with the estimated change in poly(A)-tail length between *paps1-1* mutants and wild type. Pearson correlation coefficients and p-values are indicated.
(PDF)

S5 Fig Analysis of base composition and hexamer-motif overrepresentation in 3’ UTRs. (A) Base composition in 200 bp surrounding the polyadenylation site of the 1000 loci giving rise to the transcripts with the lowest p-values for a change in poly(A)-tail length in *paps1-1* mutants (left) and in 1000 randomly chosen genes (right). (B) Distribution of variant hexamers of the canonical poly(A) signal AATAAA in all annotated *A*. *thaliana* 3’ UTRs. The two motifs marked with asterisk (AAAAAA and AAGAAA) show a statistically significant association with a poly(A)-tail length change in *paps1-1* versus wild-type (Wilcoxon rank sum test, p<0.05). Dashed red lines indicates -20 position. (C) Empirical cumulative distribution functions (CDF) of tail-length change in *paps1-1* mutants for transcripts containing the two significant motifs from (B) or the two identified significant hexamers with the largest effect size (CGCCGA and CTGACA) in the last 100 bp of their 3’ UTRs (“with”) versus the genomic background (”without”).
(PDF)

S6 Fig Estimate of ribosomal content and characterization of flow-sorted pollen. (A) Proportion of rRNA in total RNA samples of the indicated genotypes. Values represent mean ± SEM of three biological replicates each. (B) Dotplots characterizing FACS-analysis of pollen from *paps1-3*—*/ PAPS1 pLat52*::*DsRED* plants. In the left panel the pollen population is characterized by an elevated high angle scatter (SSC) and autofluorescence (observed in the 530/40 BP channel). Within this population, as shown in the middle panel, the DsRED-positive and negative pollen grains can be differentiated; numbers represent percentages of DsRED-positive and negative pollen grains, demonstrating the expected 50:50 segregation. Both pollen populations are characterized by equal FSC properties. The top population seen in the panel on the right consists of a small portion of pollen aggregates, captured by a larger time-of-flight (Pulse Width), which were excluded from the sorted population in order to maximize purity.
(PDF)

S7 Fig Absolute seedling fresh weight and root length under oxidative stress. (A,B) Absolute values for seedling fresh weight (A) and root length (B) are shown for the indicated genotypes on medium without (black) and with 40 mM methyl-viologen (grey). Values are mean ± SEM, and correspond to the experiment shown in Fig 4C and 4D.
(PDF)

S1 Table RNA-seq statistics. Counts of reads falling in the indicated categories are given for the 16 samples resulting from fractionation of 8 total-RNA starting samples.
(XLSX)

S2 Table List of MapMan categories with significantly different poly(A)-tail lengths than the transcriptome-wide background in wild type. Median(log2FC): median of the log2-ratio of fpkm values from the short- and long-tail fractions from L*er* wild-type; WT p-value: uncorrected p-value of Wilcoxon rank-sum test; WT BH p-value: p-value of Wilcoxon rank-sum test with Benjamini-Hochberg correction
(XLSX)

S3 Table Estimated changes in poly(A)-tail length in *paps1-1* mutants versus wild type. log2FC(paps1.l/s / Ler.l/s): log2 of the ratio of (long/short ratio in *paps1*) to (long/short ratio in L*er*); negative values indicate shorter poly(A)-tail in *paps1-1* mutants; log2CPM: log2-transformed counts per million (CPM) transcript abundance estimates; BH p-value: Benjamini-Hochberg corrected p-value; genes highlighted in yellow are tested in S3C Fig.
(XLSX)

S4 Table List of MapMan categories with significantly different poly(A)-tail length changes in *paps1-1* versus wild type than the transcriptome-wide background. Median(log2FC): median of the log2 fold-change of long/short ratios between *paps1-1* mutant and wild-type samples over the category in question; WT p-value: uncorrected p-value of Wilcoxon rank-sum test; WT BH p-value: p-value of Wilcoxon rank-sum test with Benjamini-Hochberg correction
(XLSX)

S5 Table Association test of all possible hexamers with a change in poly(A)-tail length in *paps1* mutants versus wild type. enrichment: log2 of mean tail-length changes for genes having the motif vs. mean tail-length changes for genes not having the motif within the last 100 bp of their 3’ UTR; wt.p.value: uncorrected p-value of a Wilcoxon rank-sum test comparing tail-length changes between *paps1* mutant and wild type for transcripts with and without the hexamer in question in their 3' UTR; BH.wt.p.value: Benjamini-Hochberg corrected p-values of the above tests
(XLSX)

S6 Table Overlap of the top 400 genes with an altered poly(A)-tail length in *paps1* mutants based on p-value with published microarray experiments as determined by MASTA. Overlap: number of genes found in the overlapping set. Yellow shading indicates experiments involving modulation of the redox balance, found within the top 3% overlapping experiments.
(XLSX)

S7 Table Behavior of overlapping genes from *tAPX*-overexpression experiment regarding tail-length change in *paps1-1* mutants. log2FC(paps1.l/s / Ler.l/s): log2 of the ratio of (long/short ratio in *paps1*) to (long/short ratio in L*er*), negative values indicate shorter poly(A)-tail in *paps1-1* mutants; logCPM: log2(counts per million); BH p-value: Benjamini-Hochberg corrected p-value; oxtAPX-wt: change in gene expression in *tAPX* overexpression line (1, upregulated; -1, downregulated)
(XLSX)

S8 Table Association of individual genes to co-expression modules. List of microarray probe sets and associated gene models, and the module the gene has been assigned to in the co-expression analysis.
(XLSX)

S9 Table Behavior of genes in the different co-expression modules regarding poly(A)-tail length change and total-RNA abundance change in *paps1* mutants. Mean (paps1 l/s vs Ler l/s), Median (paps1 l/s vs Ler l/s): Mean and median of predicted poly(A)-tail length change of genes in the given module in *paps1* mutant versus L*er* wild-type; Mean (paps1 vs Ler), Median (paps1 vs Ler): Mean and median of total RNA-abundance change of genes in the given module in *paps1* mutant versus L*er* wild-type.
(XLSX)

S10 Table Unique microarray experiments used for co-expression analysis. List of unique microarray experiments used for the co-expression analysis. If the data set is available from more than one database, the source used for the analysis presented here is indicated.
(XLSX)

## References

[1] Eckmann CR, Rammelt C, Wahle E. Control of poly(A) tail length. Wiley Interdiscip Rev RNA. 2011; 2: 348–361. 10.1002/wrna.56 21957022

[2] Hunt AG. Messenger RNA 3' end formation in plants. Curr Top Microbiol Immunol. 2008; 326: 151–177. 18630752 10.1007/978-3-540-76776-3_9

[3] Millevoi S, Vagner S. Molecular mechanisms of eukaryotic pre-mRNA 3' end processing regulation. Nucleic Acids Res. 2010; 38: 2757–2774. 10.1093/nar/gkp1176 20044349 PMC2874999

[4] Fuke H, Ohno M. Role of poly (A) tail as an identity element for mRNA nuclear export. Nucleic Acids Res. 2008; 36: 1037–1049. 18096623 10.1093/nar/gkm1120 PMC2241894

[5] Dower K, Kuperwasser N, Merrikh H, Rosbash M. A synthetic A tail rescues yeast nuclear accumulation of a ribozyme-terminated transcript. RNA. 2004; 10: 1888–1899. 15547135 10.1261/rna.7166704 PMC1370677

[6] Dunn EF, Hammell CM, Hodge CA, Cole CN. Yeast poly(A)-binding protein, Pab1, and PAN, a poly(A) nuclease complex recruited by Pab1, connect mRNA biogenesis to export. Genes Dev. 2005; 19: 90–103. 15630021 10.1101/gad.1267005 PMC540228

[7] Sonenberg N, Hinnebusch AG. Regulation of translation initiation in eukaryotes: mechanisms and biological targets. Cell. 2009; 136: 731–745. 10.1016/j.cell.2009.01.042 19239892 PMC3610329

[8] Weill L, Belloc E, Bava FA, Mendez R. Translational control by changes in poly(A) tail length: recycling mRNAs. Nat Struct Mol Biol. 2012; 19: 577–585. 10.1038/nsmb.2311 22664985

[9] Preiss T, Muckenthaler M, Hentze MW. Poly(A)-tail-promoted translation in yeast: implications for translational control. RNA. 1998; 4: 1321–1331. 9814754 10.1017/s1355838298980669 PMC1369706

[10] Kojima S, Sher-Chen EL, Green CB. Circadian control of mRNA polyadenylation dynamics regulates rhythmic protein expression. Genes Dev. 2012; 26: 2724–2736. 10.1101/gad.208306.112 23249735 PMC3533077

[11] Anderson JS, Parker RP. The 3' to 5' degradation of yeast mRNAs is a general mechanism for mRNA turnover that requires the SKI2 DEVH box protein and 3' to 5' exonucleases of the exosome complex. The EMBO journal. 1998; 17: 1497–1506. 9482746 10.1093/emboj/17.5.1497 PMC1170497

[12] Decker CJ, Parker R. A turnover pathway for both stable and unstable mRNAs in yeast: evidence for a requirement for deadenylation. Genes & development. 1993; 7: 1632–1643. 8393418 10.1101/gad.7.8.1632

[13] Hsu CL, Stevens A. Yeast cells lacking 5'—>3' exoribonuclease 1 contain mRNA species that are poly(A) deficient and partially lack the 5' cap structure. Molecular and cellular biology. 1993; 13: 4826–4835. 8336719 10.1128/mcb.13.8.4826-4835.1993 PMC360109

[14] Parker R. RNA degradation in Saccharomyces cerevisae. Genetics. 2012; 191: 671–702. 10.1534/genetics.111.137265 22785621 PMC3389967

[15] Eckhart VM, Geber MA. Character variation and geographic range in Clarkia xantiana (Onagraceae): breeding system and phenology distinguish two common subspecies. Madroño. 2000 46: 117–125.

[16] Meijer HA, Bushell M, Hill K, Gant TW, Willis AE, Jones P, et al A novel method for poly(A) fractionation reveals a large population of mRNAs with a short poly(A) tail in mammalian cells. Nucleic Acids Res. 2007; 35: e132 17933768 10.1093/nar/gkm830 PMC2095794

[17] Subtelny AO, Eichhorn SW, Chen GR, Sive H, Bartel DP. Poly(A)-tail profiling reveals an embryonic switch in translational control. Nature. 2014; 508: 66–71. 10.1038/nature13007 24476825 PMC4086860

[18] Benoit B, Mitou G, Chartier A, Temme C, Zaessinger S, Wahle E, et al An essential cytoplasmic function for the nuclear poly(A) binding protein, PABP2, in poly(A) tail length control and early development in Drosophila. Developmental cell. 2005; 9: 511–522. 16198293 10.1016/j.devcel.2005.09.002

[19] Cui J, Sartain CV, Pleiss JA, Wolfner MF. Cytoplasmic polyadenylation is a major mRNA regulator during oogenesis and egg activation in Drosophila. Developmental biology. 2013; 383: 121–131. 10.1016/j.ydbio.2013.08.013 23978535 PMC3821703

[20] Juge F, Zaessinger S, Temme C, Wahle E, Simonelig M. Control of poly(A) polymerase level is essential to cytoplasmic polyadenylation and early development in Drosophila. The EMBO journal. 2002; 21: 6603–6613. 12456666 10.1093/emboj/cdf633 PMC136937

[21] Kashiwabara S, Noguchi J, Zhuang T, Ohmura K, Honda A, Sugiura S, et al Regulation of spermatogenesis by testis-specific, cytoplasmic poly(A) polymerase TPAP. Science. 2002; 298: 1999–2002. 12471261 10.1126/science.1074632

[22] Kim KW, Wilson TL, Kimble J. GLD-2/RNP-8 cytoplasmic poly(A) polymerase is a broad-spectrum regulator of the oogenesis program. Proceedings of the National Academy of Sciences of the United States of America. 2010; 107: 17445–17450. 10.1073/pnas.1012611107 20855596 PMC2951458

[23] Suh N, Jedamzik B, Eckmann CR, Wickens M, Kimble J. The GLD-2 poly(A) polymerase activates gld-1 mRNA in the Caenorhabditis elegans germ line. Proceedings of the National Academy of Sciences of the United States of America. 2006; 103: 15108–15112. 17012378 10.1073/pnas.0607050103 PMC1622784

[24] Gohin M, Fournier E, Dufort I, Sirard M-A. Discovery, identification and sequence analysis of RNAs selected for very short or long poly A tail in immature bovine oocytes. Molecular human reproduction. 2014; 20: 127–138. 10.1093/molehr/gat080 24233545

[25] Charlesworth A, Meijer HA, de Moor CH. Specificity factors in cytoplasmic polyadenylation. Wiley Interdiscip Rev RNA. 2013; 4: 437–461. 10.1002/wrna.1171 23776146 PMC3736149

[26] Mellman DL, Gonzales ML, Song C, Barlow CA, Wang P, Kendziorski C, et al A PtdIns4,5P2-regulated nuclear poly(A) polymerase controls expression of select mRNAs. Nature. 2008; 451: 1013–1017. 10.1038/nature06666 18288197

[27] Kuhn U, Gundel M, Knoth A, Kerwitz Y, Rudel S, Wahle E. Poly(A) tail length is controlled by the nuclear poly(A)-binding protein regulating the interaction between poly(A) polymerase and the cleavage and polyadenylation specificity factor. The Journal of biological chemistry. 2009; 284: 22803–22814. 10.1074/jbc.M109.018226 19509282 PMC2755688

[28] Wahle E. A novel poly(A)-binding protein acts as a specificity factor in the second phase of messenger RNA polyadenylation. Cell. 1991; 66: 759–768. 1878970 10.1016/0092-8674(91)90119-j

[29] Hunt AG, Xu R, Addepalli B, Rao S, Forbes KP, Meeks LR, et al Arabidopsis mRNA polyadenylation machinery: comprehensive analysis of protein-protein interactions and gene expression profiling. BMC Genomics. 2008; 9: 220 10.1186/1471-2164-9-220 18479511 PMC2391170

[30] Meeks LR, Addepalli B, Hunt AG. Characterization of genes encoding poly(A) polymerases in plants: evidence for duplication and functional specialization. PLoS One. 2009; 4: e8082 10.1371/journal.pone.0008082 19956626 PMC2778134

[31] Trost G, Vi SL, Czesnick H, Lange P, Holton N, Giavalisco P, et al Arabidopsis poly(A) polymerase PAPS1 limits founder-cell recruitment to organ primordia and suppresses the salicylic acid-independent immune response downstream of EDS1/PAD4. Plant J. 2013. 10.1111/tpj.12421 24372773

[32] Vi SL, Trost G, Lange P, Czesnick H, Rao N, Lieber D, et al Target specificity among canonical nuclear poly(A) polymerases in plants modulates organ growth and pathogen response. Proc Natl Acad Sci U S A. 2013; 110: 13994–13999. 10.1073/pnas.1303967110 23918356 PMC3752211

[33] Shcherbik N, Wang M, Lapik YR, Srivastava L, Pestov DG. Polyadenylation and degradation of incomplete RNA polymerase I transcripts in mammalian cells. EMBO Rep. 2010; 11: 106–111. 10.1038/embor.2009.271 20062005 PMC2828747

[34] Win TZ, Draper S, Read RL, Pearce J, Norbury CJ, Wang SW. Requirement of fission yeast Cid14 in polyadenylation of rRNAs. Mol Cell Biol. 2006; 26: 1710–1721. 16478992 10.1128/MCB.26.5.1710-1721.2006 PMC1430263

[35] Janicke A, Vancuylenberg J, Boag PR, Traven A, Beilharz TH. ePAT: a simple method to tag adenylated RNA to measure poly(A)-tail length and other 3' RACE applications. RNA. 2012; 18: 1289–1295. 10.1261/rna.031898.111 22543866 PMC3358650

[36] Thomas PE, Wu XH, Liu M, Gaffney B, Ji GL, Li QSQ, et al Genome-Wide Control of Polyadenylation Site Choice by CPSF30 in Arabidopsis. Plant Cell. 2012; 24: 4376–4388. 10.1105/tpc.112.096107 23136375 PMC3531840

[37] Sherstnev A, Duc C, Cole C, Zacharaki V, Hornyik C, Ozsolak F, et al Direct sequencing of Arabidopsis thaliana RNA reveals patterns of cleavage and polyadenylation. Nat Struct Mol Biol. 2012; 19: 845–852. 10.1038/nsmb.2345 22820990 PMC3533403

[38] Narsai R, Howell KA, Millar AH, O'Toole N, Small I, Whelan J. Genome-wide analysis of mRNA decay rates and their determinants in Arabidopsis thaliana. The Plant cell. 2007; 19: 3418–3436. 18024567 10.1105/tpc.107.055046 PMC2174890

[39] Amrani N, Ghosh S, Mangus DA, Jacobson A. Translation factors promote the formation of two states of the closed-loop mRNP. Nature. 2008; 453: 1276–1280. 10.1038/nature06974 18496529 PMC2587346

[40] Moritz M, Paulovich AG, Tsay YF, Woolford JL Jr. Depletion of yeast ribosomal proteins L16 or rp59 disrupts ribosome assembly. J Cell Biol. 1990; 111: 2261–2274. 2277060 10.1083/jcb.111.6.2261 PMC2116383

[41] Warner JR, Udem SA. Temperature sensitive mutations affecting ribosome synthesis in Saccharomyces cerevisiae. J Mol Biol. 1972; 65: 243–257. 4557193 10.1016/0022-2836(72)90280-x

[42] Francis KE, Lam SY, Harrison BD, Bey AL, Berchowitz LE, Copenhaver GP. Pollen tetrad-based visual assay for meiotic recombination in Arabidopsis. Proc Natl Acad Sci U S A. 2007; 104: 3913–3918. 17360452 10.1073/pnas.0608936104 PMC1805420

[43] Borges F, Gardner R, Lopes T, Calarco JP, Boavida LC, Slotkin RK, et al FACS-based purification of Arabidopsis microspores, sperm cells and vegetative nuclei. Plant Methods. 2012; 8: 44 10.1186/1746-4811-8-44 23075219 PMC3502443

[44] Reina-Pinto JJ, Voisin D, Teodor R, Yephremov A. Probing differentially expressed genes against a microarray database for in silico suppressor/enhancer and inhibitor/activator screens. Plant J. 2010; 61: 166–175. 10.1111/j.1365-313X.2009.04043.x 19811619

[45] Laloi C, Stachowiak M, Pers-Kamczyc E, Warzych E, Murgia I, Apel K. Cross-talk between singlet oxygen- and hydrogen peroxide-dependent signaling of stress responses in Arabidopsis thaliana. Proc Natl Acad Sci U S A. 2007; 104: 672–677. 17197417 10.1073/pnas.0609063103 PMC1766442

[46] Schwarzlander M, Fricker MD, Muller C, Marty L, Brach T, Novak J, et al Confocal imaging of glutathione redox potential in living plant cells. Journal of Microscopy. 2008; 231: 299–316. 10.1111/j.1365-2818.2008.02030.x 18778428

[47] Vranova E, Atichartpongkul S, Villarroel R, Van Montagu M, Inze D, Van Camp W. Comprehensive analysis of gene expression in Nicotiana tabacum leaves acclimated to oxidative stress. Proc Natl Acad Sci U S A. 2002; 99: 10870–10875. 12122207 10.1073/pnas.152337999 PMC125065

[48] Zhang J, Addepalli B, Yun K-Y, Hunt AG, Xu R, Rao S, et al A polyadenylation factor subunit implicated in regulating oxidative signaling in Arabidopsis thaliana. PLoS One. 2008; 3: e2410 10.1371/journal.pone.0002410 18545667 PMC2408970

[49] Langfelder P, Horvath S. WGCNA: an R package for weighted correlation network analysis. BMC Bioinformatics. 2008; 9: 559 10.1186/1471-2105-9-559 19114008 PMC2631488

[50] Beilharz TH, Preiss T. Widespread use of poly(A) tail length control to accentuate expression of the yeast transcriptome. RNA. 2007; 13: 982–997. 17586758 10.1261/rna.569407 PMC1894919

[51] Lackner DH, Beilharz TH, Marguerat S, Mata J, Watt S, Schubert F, et al A network of multiple regulatory layers shapes gene expression in fission yeast. Mol Cell. 2007; 26: 145–155. 17434133 10.1016/j.molcel.2007.03.002 PMC1885965

[52] Chang H, Lim J, Ha M, Kim VN. TAIL-seq: Genome-wide Determination of Poly(A) Tail Length and 3' End Modifications. Mol Cell. 2014; 53: 1044–1052. 10.1016/j.molcel.2014.02.007 24582499

[53] Grigull J, Mnaimneh S, Pootoolal J, Robinson MD, Hughes TR. Genome-wide analysis of mRNA stability using transcription inhibitors and microarrays reveals posttranscriptional control of ribosome biogenesis factors. Molecular and cellular biology. 2004; 24: 5534–5547. 15169913 10.1128/MCB.24.12.5534-5547.2004 PMC419893

[54] Suter L, Widmer A. Phenotypic effects of salt and heat stress over three generations in Arabidopsis thaliana. PLoS One. 2013; 8: e80819 10.1371/journal.pone.0080819 24244719 PMC3828257

[55] Sarkar D. Package ‘lattice’. 2014.

[56] Schmieder R, Lim YW, Edwards R. Identification and removal of ribosomal RNA sequences from metatranscriptomes. Bioinformatics. 2012; 28: 433–435. 10.1093/bioinformatics/btr669 22155869 PMC3268242

[57] Kim D, Pertea G, Trapnell C, Pimentel H, Kelley R, Salzberg SL. TopHat2: accurate alignment of transcriptomes in the presence of insertions, deletions and gene fusions. Genome Biol. 2013; 14: R36 10.1186/gb-2013-14-4-r36 23618408 PMC4053844

[58] Li H, Handsaker B, Wysoker A, Fennell T, Ruan J, Homer N, et al The Sequence Alignment/Map format and SAMtools. Bioinformatics. 2009; 25: 2078–2079. 10.1093/bioinformatics/btp352 19505943 PMC2723002

[59] Anders S, Pyl PT, Huber W. HTSeq–A Python framework to work with high-throughput sequencing data. biorxiv. 2014. 10.1093/bioinformatics/btu638 PMC4287950 25260700

[60] Trapnell C, Williams BA, Pertea G, Mortazavi A, Kwan G, van Baren MJ, et al Transcript assembly and quantification by RNA-Seq reveals unannotated transcripts and isoform switching during cell differentiation. Nature Biotechnology. 2010; 28: 511–U174. 10.1038/nbt.1621 20436464 PMC3146043

[61] Gentleman RC, Carey VJ, Bates DM, Bolstad B, Dettling M, Dudoit S, et al Bioconductor: open software development for computational biology and bioinformatics. Genome Biol. 2004; 5: R80 15461798 10.1186/gb-2004-5-10-r80 PMC545600

[62] Robinson MD, McCarthy DJ, Smyth GK. edgeR: a Bioconductor package for differential expression analysis of digital gene expression data. Bioinformatics. 2010; 26: 139–140. 10.1093/bioinformatics/btp616 19910308 PMC2796818

[63] Benjamini Y, Hochberg Y. Controlling the False Discovery Rate: A Practical and Powerful Approach to Multiple Testing. Journal of the Royal Statistical Society Series B (Methodological). 1995; 57: 289–300.

[64] Robinson MD, Oshlack A. A scaling normalization method for differential expression analysis of RNA-seq data. Genome Biol. 2010; 11: R25 10.1186/gb-2010-11-3-r25 20196867 PMC2864565

[65] Thimm O, Blasing O, Gibon Y, Nagel A, Meyer S, Kruger P, et al MAPMAN: a user-driven tool to display genomics data sets onto diagrams of metabolic pathways and other biological processes. Plant J. 2004; 37: 914–939. 14996223 10.1111/j.1365-313x.2004.02016.x

[66] Barrett T, Wilhite SE, Ledoux P, Evangelista C, Kim IF, Tomashevsky M, et al NCBI GEO: archive for functional genomics data sets—update. Nucleic Acids Res. 2013; 41: D991–995. 10.1093/nar/gks1193 23193258 PMC3531084

[67] Rustici G, Kolesnikov N, Brandizi M, Burdett T, Dylag M, Emam I, et al ArrayExpress update—trends in database growth and links to data analysis tools. Nucleic Acids Res. 2013; 41: D987–990. 10.1093/nar/gks1174 23193272 PMC3531147

[68] Craigon DJ, James N, Okyere J, Higgins J, Jotham J, May S. NASCArrays: a repository for microarray data generated by NASC's transcriptomics service. Nucleic Acids Res. 2004; 32: D575–577. 14681484 10.1093/nar/gkh133 PMC308867

[69] Gautier L, Cope L, Bolstad BM, Irizarry RA. affy—analysis of Affymetrix GeneChip data at the probe level. Bioinformatics. 2004; 20: 307–315. 14960456 10.1093/bioinformatics/btg405

