# Supplementary figures and images for: Genome-Wide Analysis of PAPS1-Dependent Polyadenylation Identifies Novel Roles for Functionally Specialized Poly(A) Polymerases in Arabidopsis thaliana
Source: PLoS Genet. 2015 Aug 25;11(8):e1005474. doi: 10.1371/journal.pgen.1005474 (PMC4549238; doi:10.1371/journal.pgen.1005474)

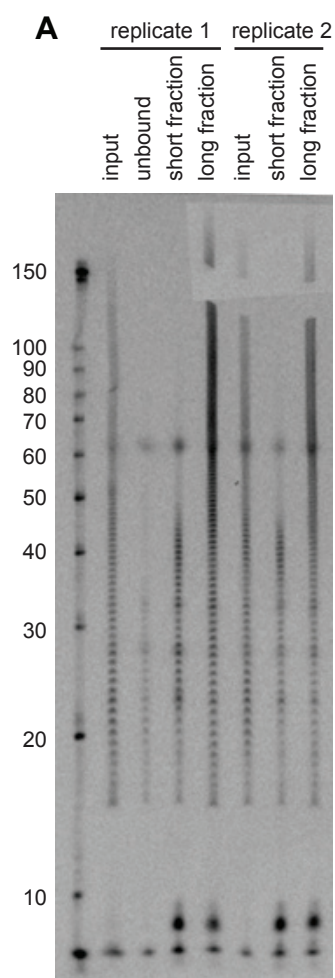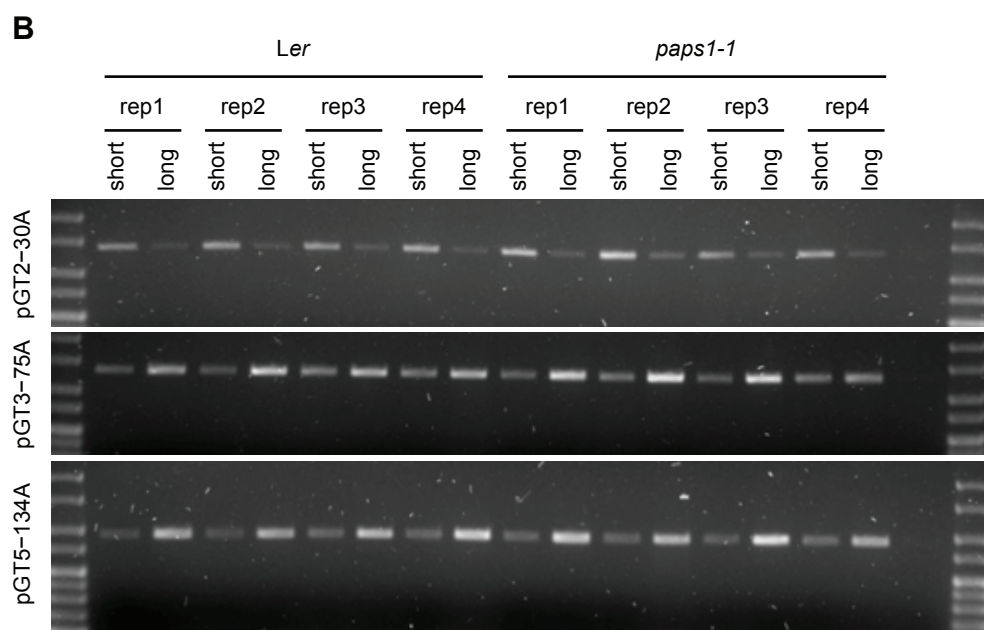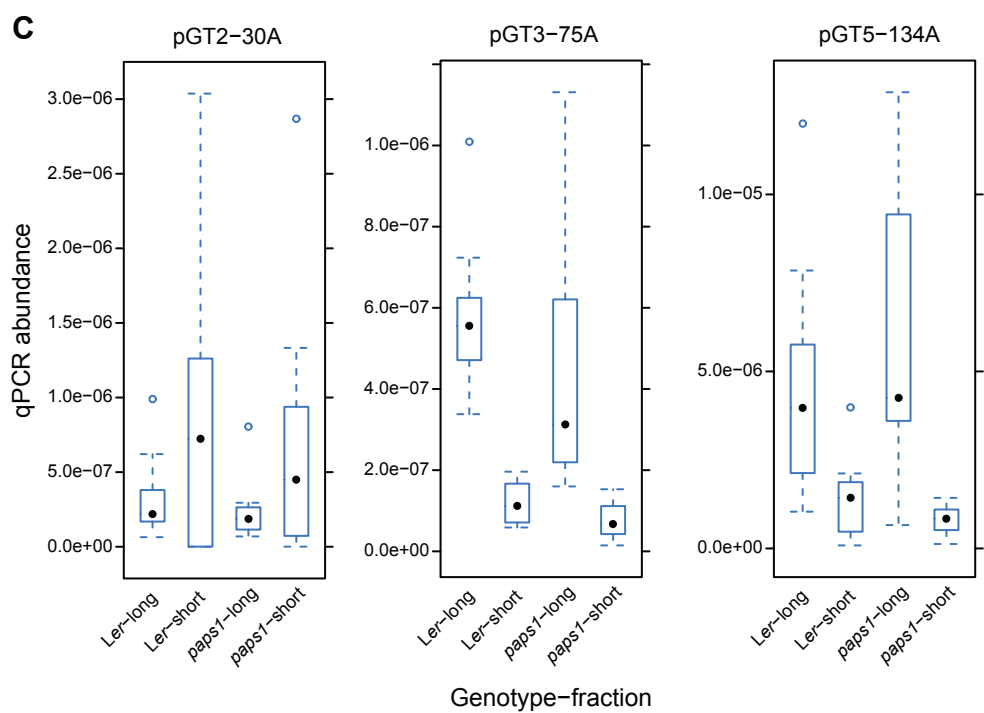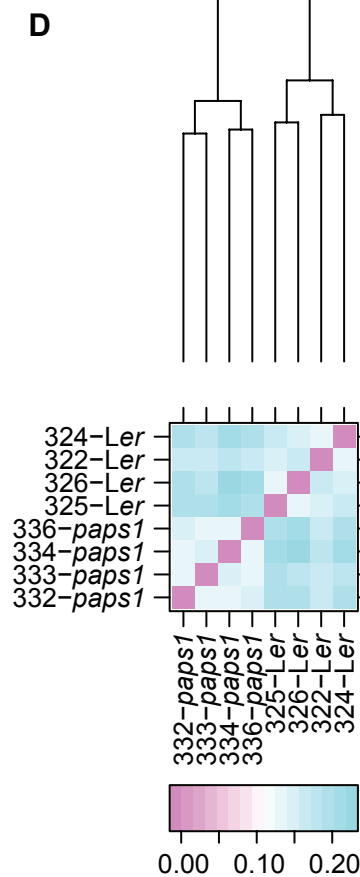

Supplement: S1 Fig — (A) Bulk poly(A)-tail analysis of the input and the unbound, short-tail and long-tail fractions from two biological replicates of wild-type seedlings. RNA was treated as described in the legend to Fig 1. Fragment sizes in nucleotides are indicated on the left based on a labeled RNA-size marker. The discontinuity at the top of the four right-most lanes is due to a tear in the gel. (B) RT-PCR products representing the three spike-in controls from the indicated fractions were separared by agarose gel electrophoresis. (C) Abundance estimates of the three spike-in control RNAs from qRT-PCR of the short- and long-tail fractions. Box-plots show the distribution of values from four biological replicates each. Filled black dots indicate the median value; open blue circles are outliers as determined using the R/lattice boxplot.stats function. (D) Clustering of samples based on fpkm ratios from the long-tail and the short-tail fractions (long/short ratio) after filtering out lowly expressed genes (fpkm < 0.5) and using 1 –correlation between each two samples as distance. Biological replicates cluster together well, when not considering lowly expressed genes subject to higher measurement variability using RNA-seq. (PDF) [file pgen.1005474.s001.pdf]

**A**

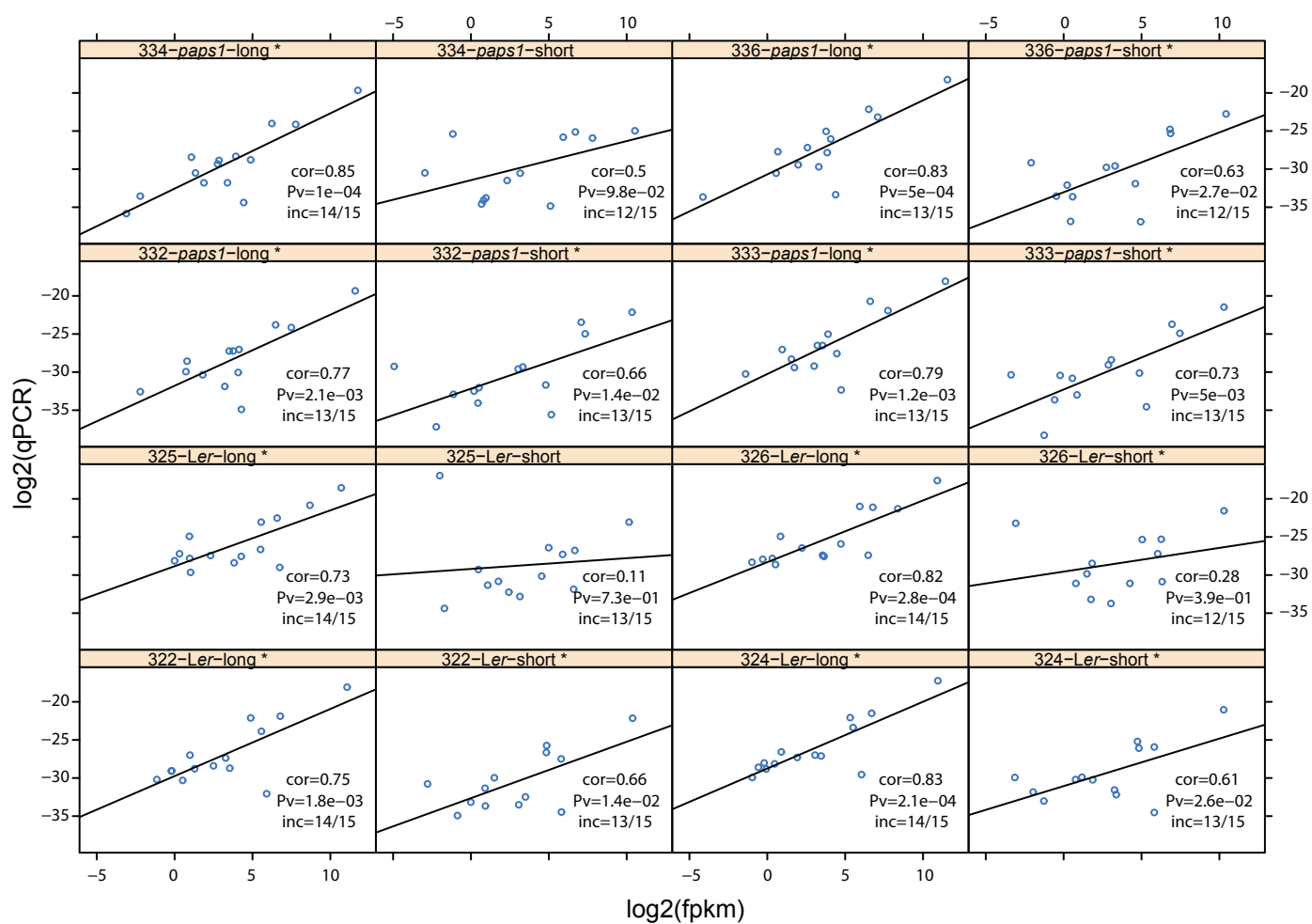

**B**

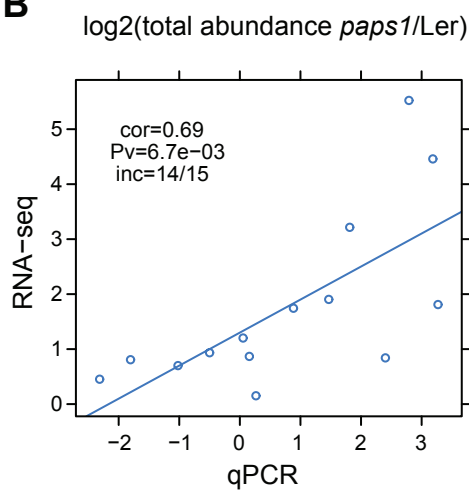

Supplement: S2 Fig — (A) Correlation plots of abundance estimates from qPCR with those from RNA-seq for 15 transcripts from the indicated fractions. Pearson correlation coefficients, p-values and the number of included transcripts are shown in each panel. If fewer than 15 transcripts were included, this was because qPCR detection was unreliable. Asterisk indicates significant correlation at p<0.05. (B) Correlation of estimated fold-changes in total transcript abundance derived from qRT-PCR or from RNA-seq for the 15 transcripts from (A). qRT-PCR was performed on three biological-replicate RNA samples from seedlings grown in parallel to those used for the RNA fractionation. For the RNA-seq based estimates fpkm values were used to also include lowly expressed genes. The 15th gene excluded from the figure has no mapped sequencing reads in any of the three Ler samples; consistently, qRT-PCR also indicates a much higher abundance in paps1-1 (log2FC > 10). (PDF) [file pgen.1005474.s002.pdf]

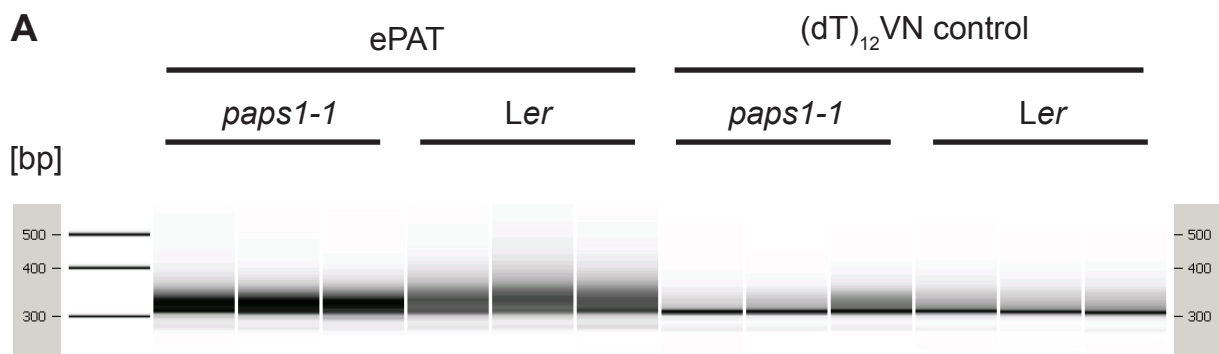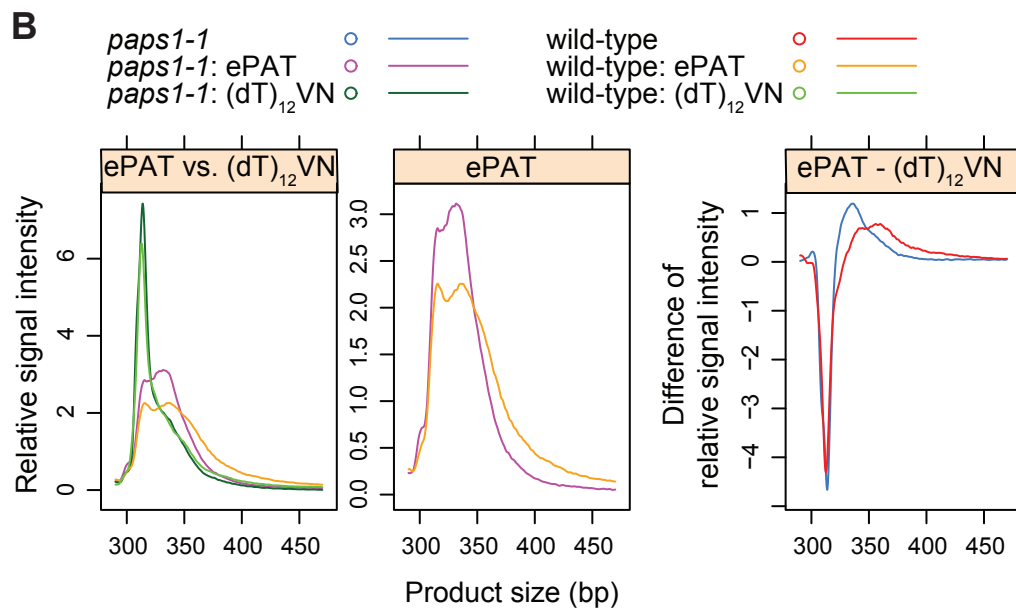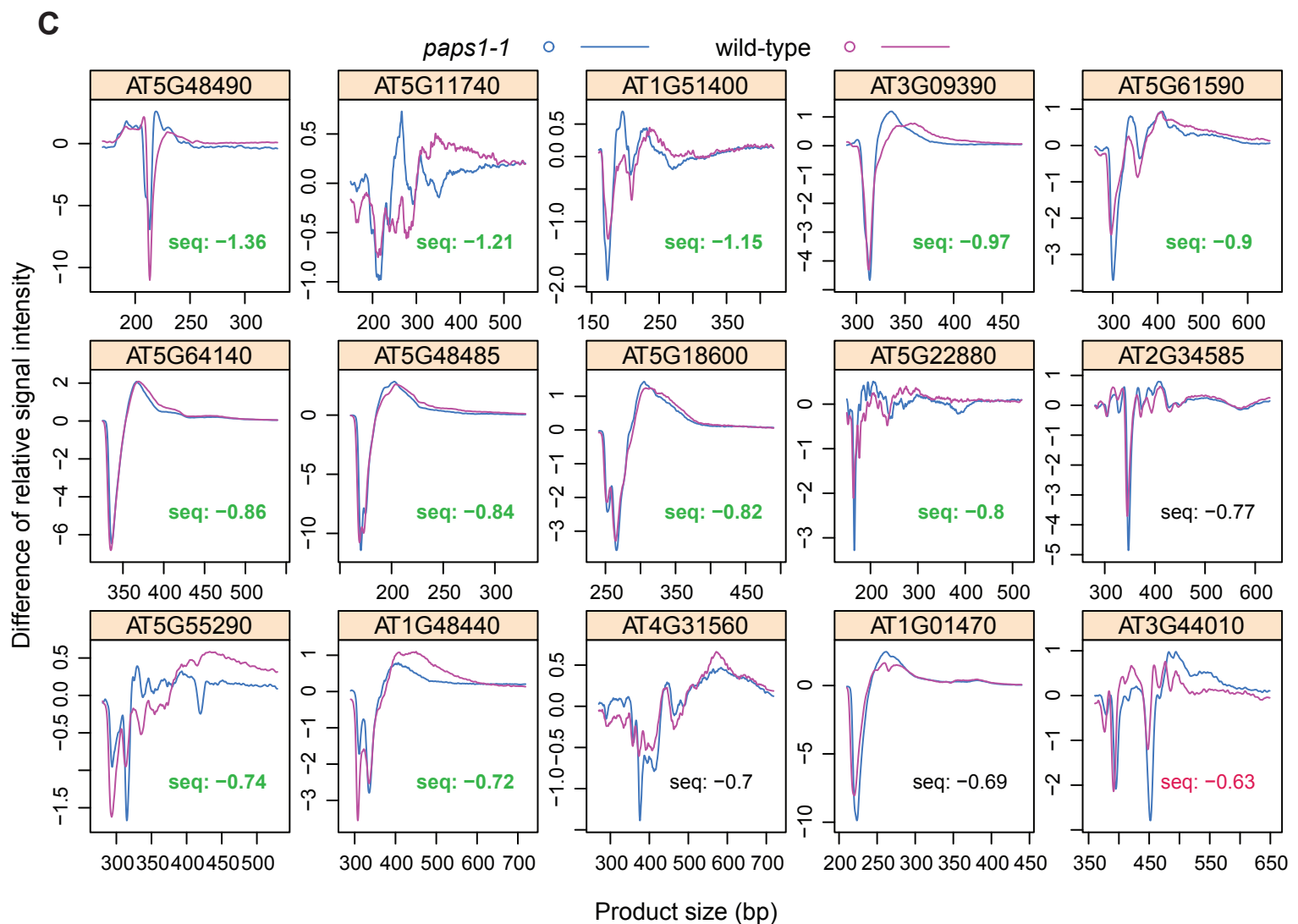

Supplement: S3 Fig — (A) Bioanalyzer results of ePAT (left) and (dT)12VN control (right) PCR products for At3g09390 mRNA from paps1-1 and Ler wild-type samples. Three biological replicates were used per genotype. Size standard is shown in bp. (B) Analysis of the results from (A). Normalized signal intensity (averaged over the three biological replicates) is shown for the ePAT and control products from both genotypes (left) and for ePAT only (middle). Right panel shows the difference in relative signal intensities between the ePAT and control products. The broad shoulder to the right of the dip at 315 bp represents the poly(A) tail. The shift of the wild-type curve (red) towards longer products relative to the mutant curve (blue) indicates the longer poly(A) tail in the wild-type samples. (C) Differences of relative signal intensities between ePAT and control samples as determined in the right panel of (B) are shown for 15 transcripts. Numbers in the panels indicate the predicted log2-fold change of the poly(A)-tail length between paps1-1 and wild-type samples based on the fractionation/RNA-seq approach. Green font indicates genes for which the ePAT assay indicates longer poly(A) tails from wild-type than from mutant samples (i.e. higher values for the purple than the blue curve at longer product lengths); black font indicates transcripts without a robust change, and red font denotes shorter poly(A) tails in wild-type than mutant samples. (PDF) [file pgen.1005474.s003.pdf]

**A**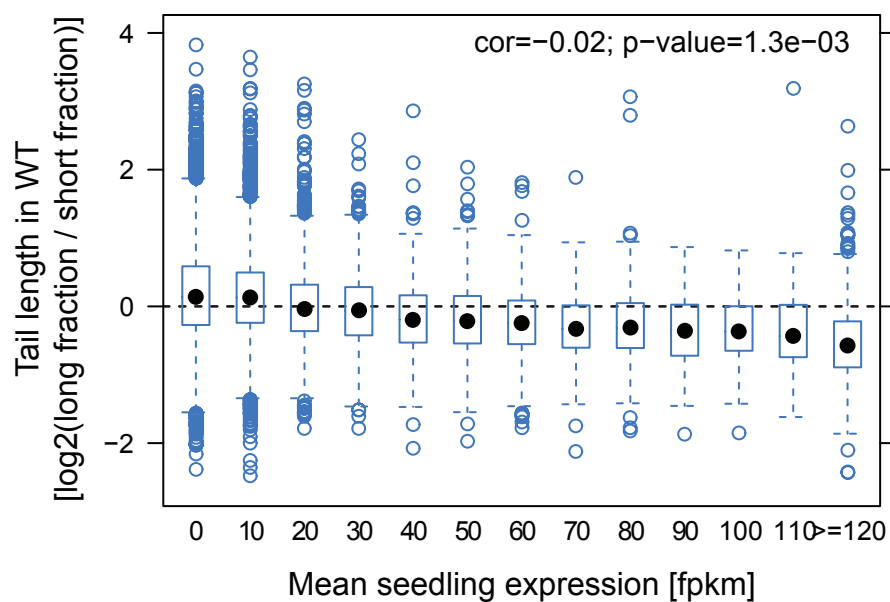**B**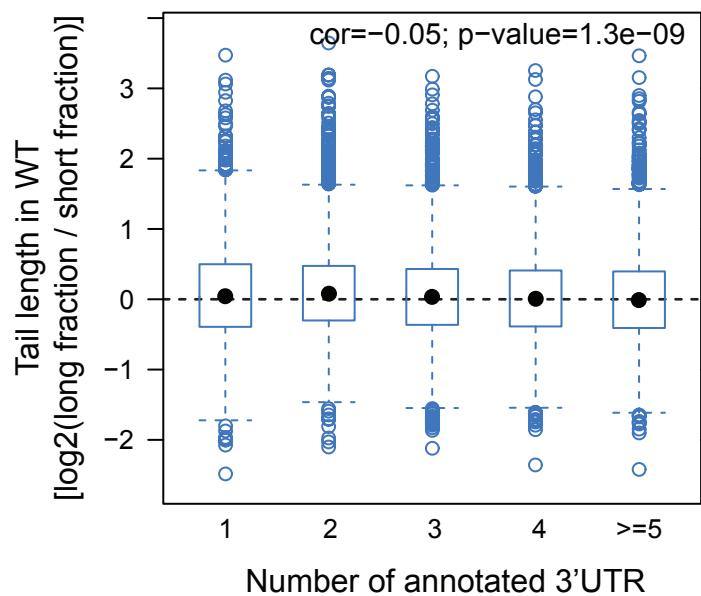**C**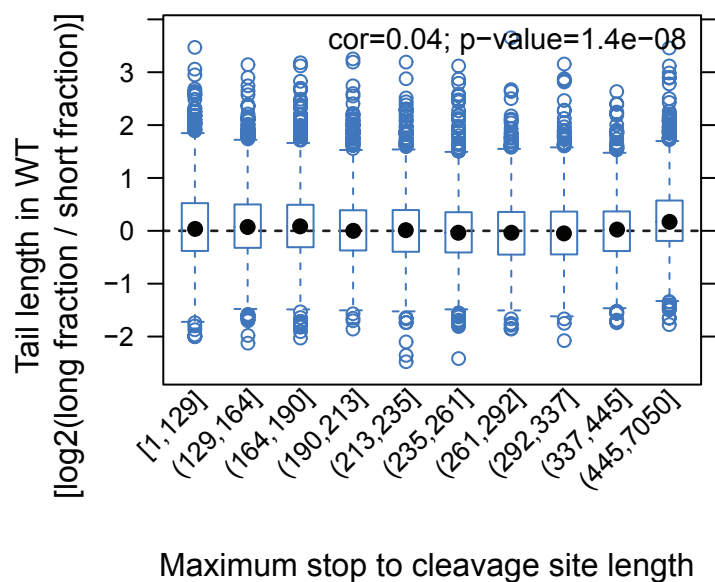**D**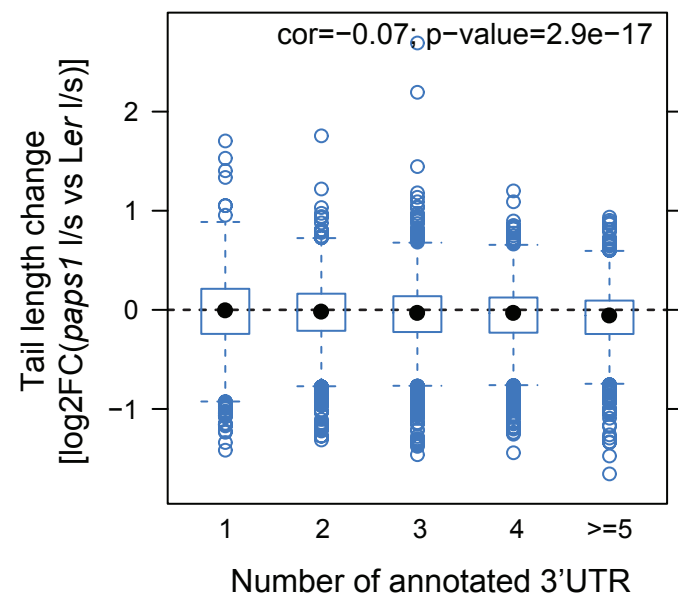**E**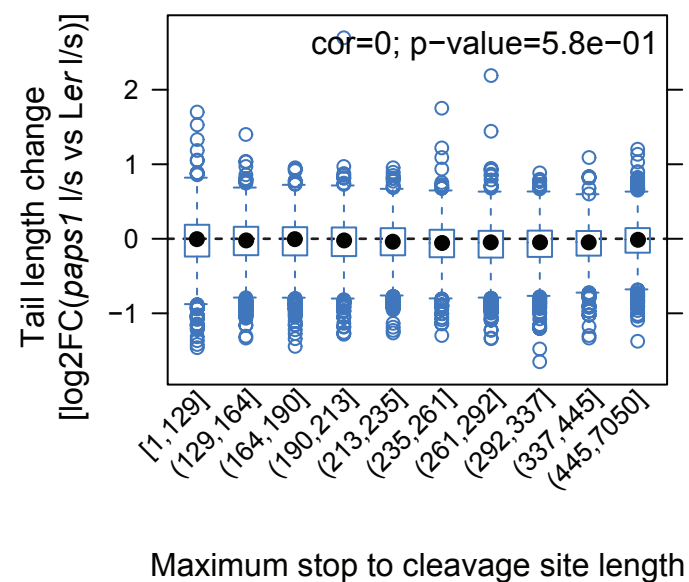

Supplement: S4 Fig — (A-C) Correlation of transcript abundance (A), number of annotated 3’ UTRs (B) and length of the 3’ UTR (C) with estimated poly(A)-tail length in wild-type. (D,E) Correlation of number of annotated 3’ UTRs (D) and length of the 3’ UTR (E) with the estimated change in poly(A)-tail length between paps1-1 mutants and wild type. Pearson correlation coefficients and p-values are indicated. (PDF) [file pgen.1005474.s004.pdf]

**A**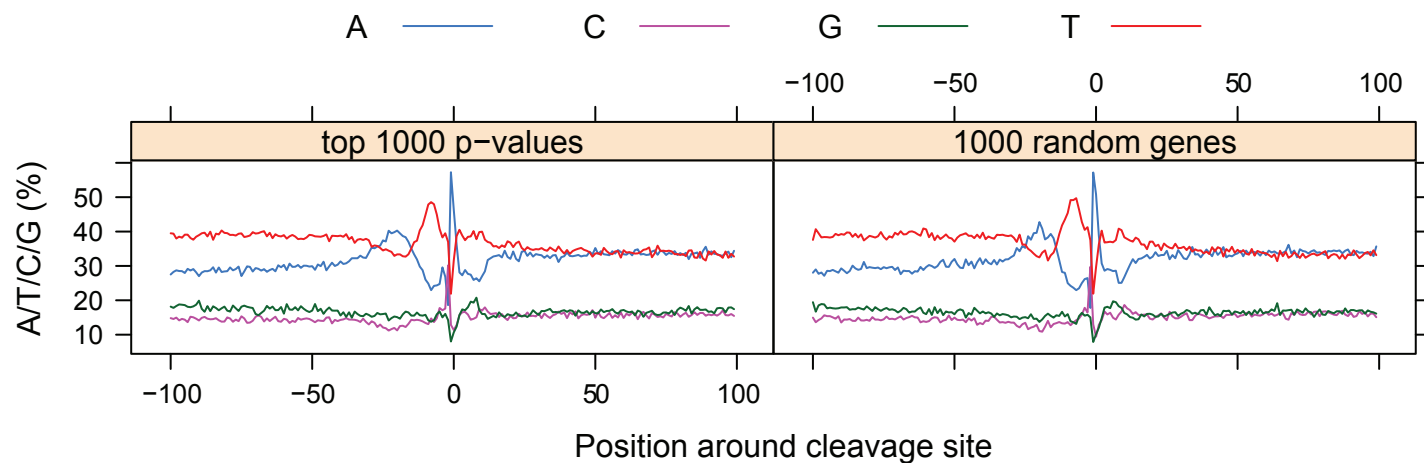**B**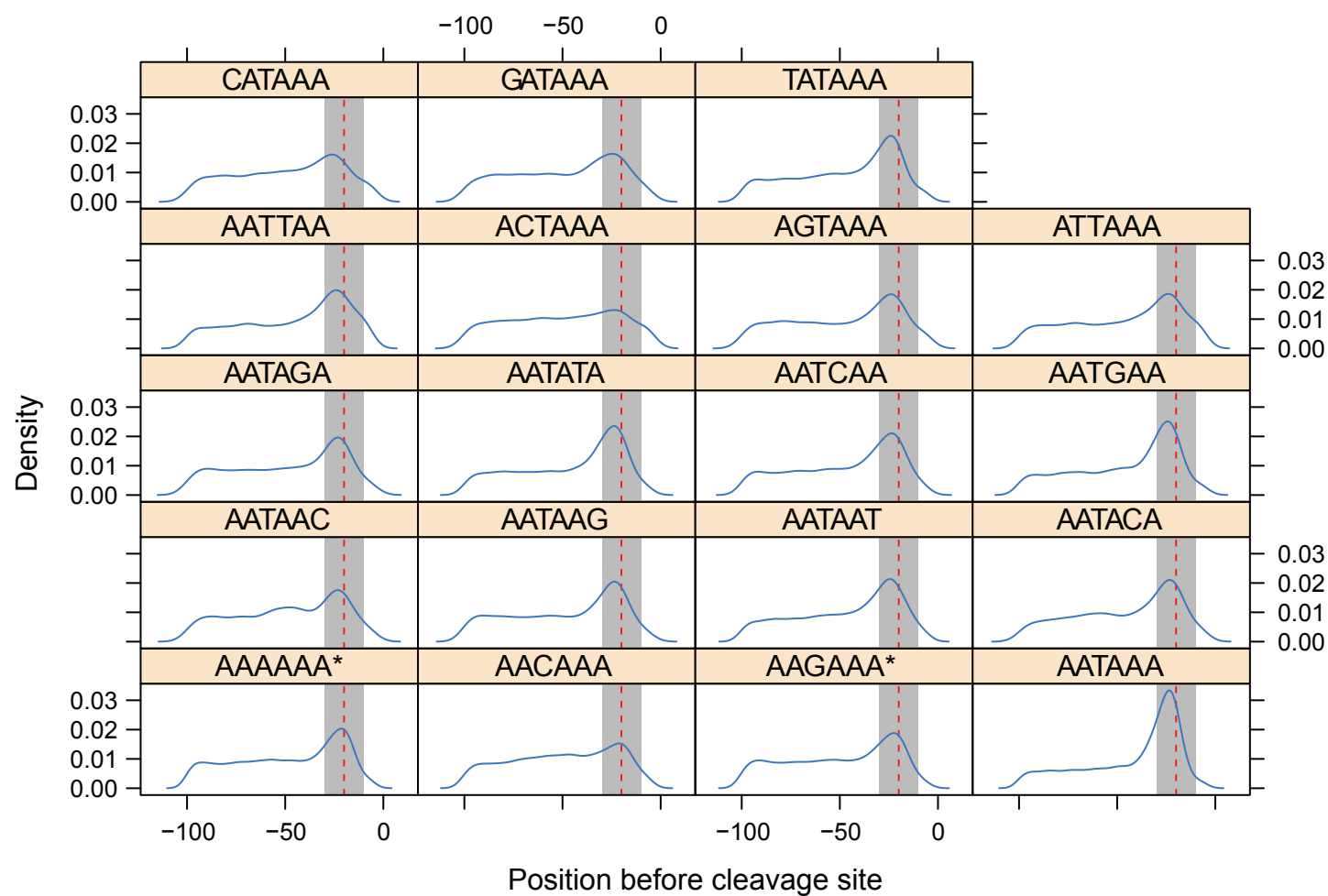**C**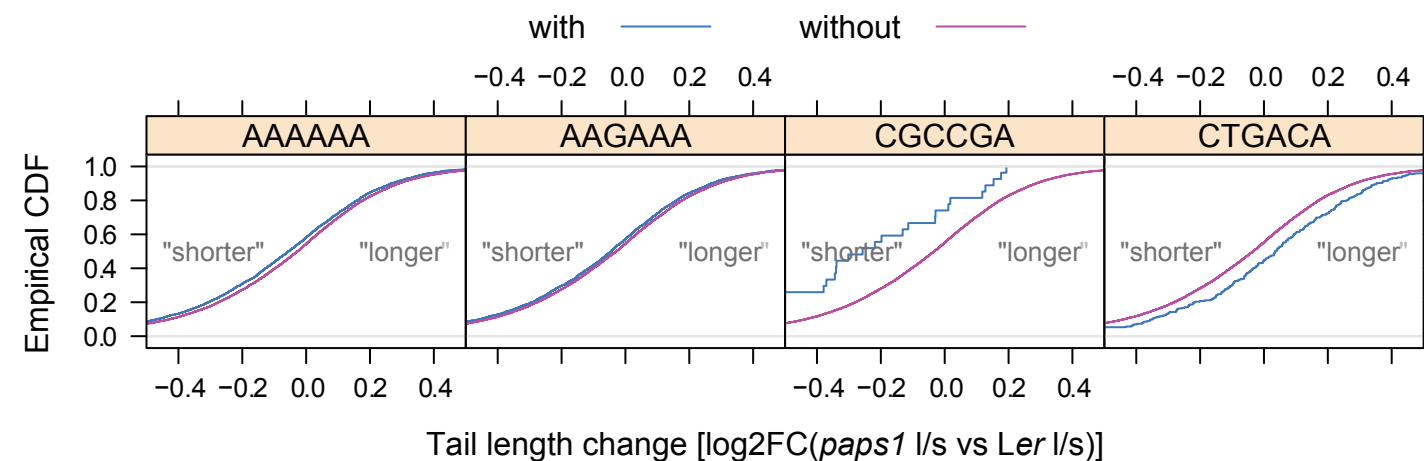

Supplement: S5 Fig — (A) Base composition in 200 bp surrounding the polyadenylation site of the 1000 loci giving rise to the transcripts with the lowest p-values for a change in poly(A)-tail length in paps1-1 mutants (left) and in 1000 randomly chosen genes (right). (B) Distribution of variant hexamers of the canonical poly(A) signal AATAAA in all annotated A. thaliana 3’ UTRs. The two motifs marked with asterisk (AAAAAA and AAGAAA) show a statistically significant association with a poly(A)-tail length change in paps1-1 versus wild-type (Wilcoxon rank sum test, p<0.05). Dashed red lines indicates -20 position. (C) Empirical cumulative distribution functions (CDF) of tail-length change in paps1-1 mutants for transcripts containing the two significant motifs from (B) or the two identified significant hexamers with the largest effect size (CGCCGA and CTGACA) in the last 100 bp of their 3’ UTRs (“with”) versus the genomic background (”without”). (PDF) [file pgen.1005474.s005.pdf]

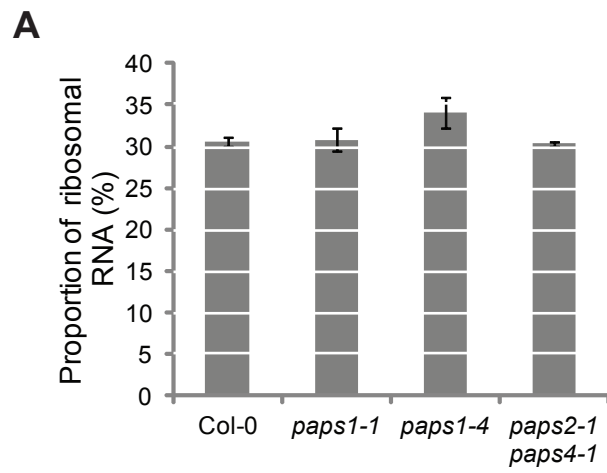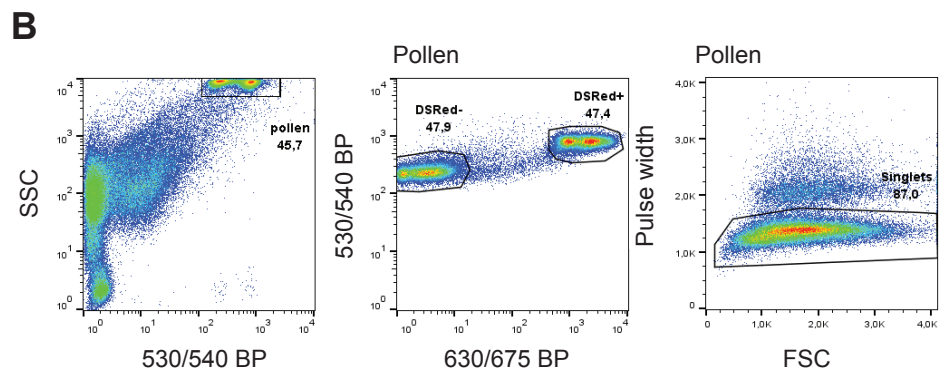

Supplement: S6 Fig — (A) Proportion of rRNA in total RNA samples of the indicated genotypes. Values represent mean ± SEM of three biological replicates each. (B) Dotplots characterizing FACS-analysis of pollen from paps1-3—/ PAPS1 pLat52::DsRED plants. In the left panel the pollen population is characterized by an elevated high angle scatter (SSC) and autofluorescence (observed in the 530/40 BP channel). Within this population, as shown in the middle panel, the DsRED-positive and negative pollen grains can be differentiated; numbers represent percentages of DsRED-positive and negative pollen grains, demonstrating the expected 50:50 segregation. Both pollen populations are characterized by equal FSC properties. The top population seen in the panel on the right consists of a small portion of pollen aggregates, captured by a larger time-of-flight (Pulse Width), which were excluded from the sorted population in order to maximize purity. (PDF) [file pgen.1005474.s006.pdf]

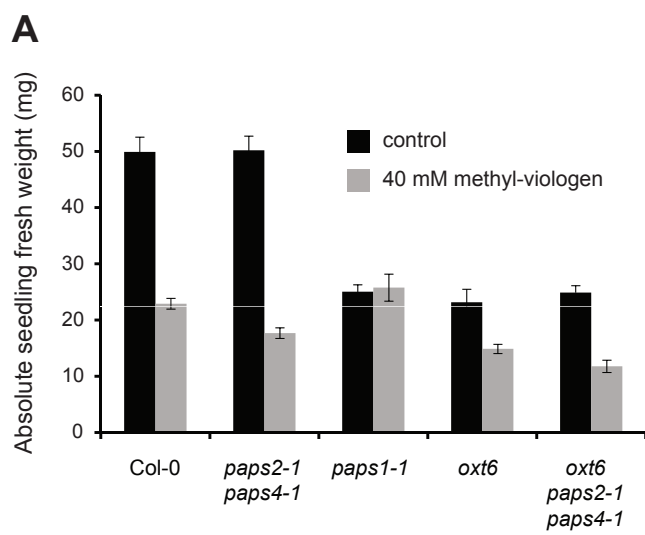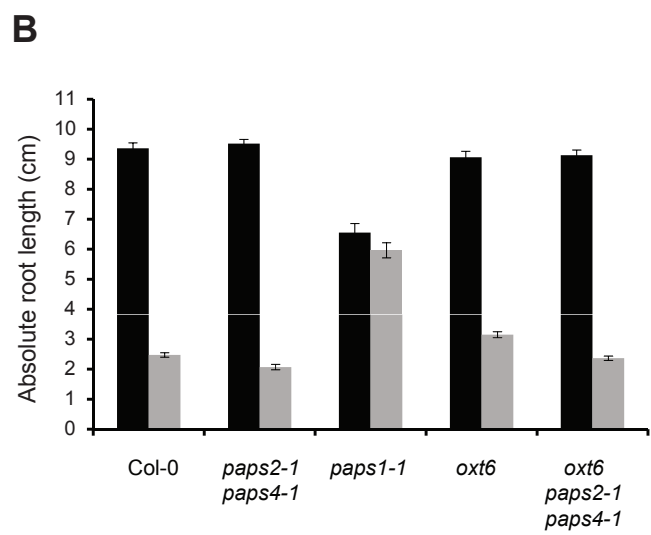

Supplement: S7 Fig — (A,B) Absolute values for seedling fresh weight (A) and root length (B) are shown for the indicated genotypes on medium without (black) and with 40 mM methyl-viologen (grey). Values are mean ± SEM, and correspond to the experiment shown in Fig 4C and 4D. (PDF) [file pgen.1005474.s007.pdf]
